# Genome-wide association study and genomic selection for yield and related traits in soybean

**DOI:** 10.1371/journal.pone.0255761

**Published:** 2021-08-13

**Authors:** Waltram Ravelombola, Jun Qin, Ainong Shi, Qijian Song, Jin Yuan, Fengmin Wang, Pengyin Chen, Long Yan, Yan Feng, Tiantian Zhao, Yaning Meng, Kexin Guan, Chunyan Yang, Mengchen Zhang

**Affiliations:** 1 The Key Laboratory of Crop Genetics and Breeding of Hebei Province, National Soybean Improvement Center Shijiazhuang Sub-Center, North China Key Laboratory of Biology and Genetic Improvement of Soybean, Ministry of Agriculture, Laboratory of Crop Genetics and Breeding of Hebei Cereal & Oil Crop Institute, Hebei Academy of Agricultural and Forestry Sciences, Shijiazhuang, Hebei, China; 2 Department of Horticulture, University of Arkansas, Fayetteville, AR, United States of America; 3 Soybean Genomics and Improvement Laboratory, Beltsville Agricultural Research Center, USDA-ARS, Beltsville, MD, United States of America; 4 Fisher Delta Research Center, University of Missouri, MO, United States of America; Institute of Genetics and Developmental Biology Chinese Academy of Sciences, CHINA

## Abstract

Soybean [*Glycine max (L*.*) Merr*.] is a crop of great interest worldwide. Exploring molecular approaches to increase yield genetic gain has been one of the main challenges for soybean breeders and geneticists. Agronomic traits such as maturity, plant height, and seed weight have been found to contribute to yield. In this study, a total of 250 soybean accessions were genotyped with 10,259 high-quality SNPs postulated from genotyping by sequencing (GBS) and evaluated for grain yield, maturity, plant height, and seed weight over three years. A genome-wide association study (GWAS) was performed using a Bayesian Information and Linkage Disequilibrium Iteratively Nested Keyway (BLINK) model. Genomic selection (GS) was evaluated using a ridge regression best linear unbiased predictor (rrBLUP) model. The results revealed that 20, 31, 37, and 23 SNPs were significantly associated with maturity, plant height, seed weight, and yield, respectively; Many SNPs were mapped to previously described maturity and plant height loci (*E2*, *E4*, and *Dt1*) and a new plant height locus was mapped to chromosome 20. Candidate genes were found in the vicinity of the two SNPs with the highest significant levels associated with yield, maturity, plant height, seed weight, respectively. A 11.5-Mb region of chromosome 10 was associated with both yield and seed weight. Overall, the accuracy of GS was dependent on the trait, year, and population structure, and high accuracy indicates that these agronomic traits can be selected in molecular breeding through GS. The SNP markers identified in this study can be used to improve yield and agronomic traits through the marker-assisted selection and GS in breeding programs.

## Introduction

The success of a newly developed soybean [*Glycine max* (L.) Merr.] variety relies on a large number of characteristics including maturity, plant height, seed weight, and yield performance [1]. Soybean breeders have been focusing on improving these traits in their cultivar development. Highly heritable traits such as maturity and plant height can be selected at earlier stages, whereas complex traits such as yield are tested over many years and environments throughout the breeding pipeline. With the rapid development of genomic-related tools and DNA sequencing technology, the improvement of the agronomic and yield-related traits of interest could be performed faster.

Genome-wide association study (GWAS) and genomic selection (GS) are powerful tools to understand the genetic architecture controlling complex traits of importance in soybean. GWAS has been conducted to identify molecular markers associated with many agronomic traits in soybean [2, 3]. To date, more than 60 markers identified through GWAS have been reported to be associated with maturity in soybean [4–9]. These markers are distributed across the soybean genome but more found on chromosome 16 (https://www.soybase.org/). Candidate genes such as *Glyma11g14150*, *Glyma16g02840*, and *Glyma16g03580* were studied in detail and found to control maturity in soybean [9]. The most described loci affecting maturity in soybean are *E1-E10* and *J* [10]. Efforts towards identifying significant loci controlling plant height in soybean via GWAS have also been undertaken. Recently, 68 markers affecting plant height have been identified using GWAS [6, 7, 9, 11, 12]. Out of these, 19 were mapped on chromosome 19 (https://www.soybase.org/). A total of 11 candidate genes affecting plant height have been reported [11, 12]. A total of 95 molecular markers associated with seed weight have been previously identified in soybean, of which 12 are located on chromosome four [1, 5, 6, 8, 11–16]. Eighteen potential candidate genes have been reported for seed weight in soybean [1, 9, 11, 14]. GWAS has been proven to be efficient in identifying molecular markers associated with yield in soybean as well. Four SNPs were found to be associated with yield across multiple environments via GWAS of 219 soybean accessions [12] and the candidate genes *Glyma*.*13g073900*, *Glyma*.*06g050300*, *Glyma*.*03g169700*, and *Glyma*.*03g171900* were found in the vicinity of these SNPs. In addition, a total of 139 soybean accessions were genotyped using the BARCSoySNP6K in order to conduct GWAS for yield, reporting a total of six significant SNPs associated with yield, of which, four were located on chromosome 12 [11]. An additional study also suggested six significant loci associated with yield in soybean [5]. Copley et al. [8] reported three significant SNPs associated with yield through GWAS.

GS has allowed the estimation of the effects of all markers across the genome. The combination of the markers effect denominated genomic estimated breeding values (GEBVs) can be used to predict the performance of a line [17]. GS has been shown to outperform the traditional marker-assisted selection (MAS) in complex traits such as yield and seed weight [18]. The establishment of GS for complex traits may allow faster genetics gain per unit of time [18]. Matei et al. [19] showed that the selection cycle for yield and seed weight can be significantly reduced using GS. Previous reports showed a discrepancy regarding the accuracy of GS for complex traits in soybean. Duhnen et al. [20] reported an accuracy of 0.39 for yield in soybean, whereas Jarquín et al. [21] reported accuracy of up to 0.80 for soybean yield in an elite population consisting of 301 genotypes.

Association panels for GWASs should include a high degree of genetic diversity, and the phenotypes should be accurately characterized in various environments [22] In this research, a total of 250 soybean germplasm accessions including 60 mini core collections and 108 typical cultivars from China and 82 foreign germplasms separately from the United States, South Korea and Japan were used in GWAS and GS for yield, maturity, plant height and seed weight over 3 years. This panel can contribute towards discovering new loci of interest and confirming the previously reported ones, which will significantly enhance breeding for agronomic traits in soybean. In addition, GS-related studies in soybean still remain limited. Therefore, the objectives of this study were to investigate the population structure within a soybean panel, to perform GWAS and identify SNP markers regulating maturity, plant height, seed weight, and yield, and to assess the accuracy of GS for these traits.

## Materials and methods

### Plant materials and phenotyping

A total of 250 soybean accessions from Dr. Lijuan Qiu’s lab (Chinese Academy Of Agricultural Sciences) were used in the study (S1 Table). Soybean accessions were planted in Shijiazhuang (114°83′E, 38°03′N) in Hebei province, China with a randomized complete block design (RCBD) of three replications during the growing seasons of 2008, 2009, and 2010. A total of 90 soybean seeds were sown in three rows of 3 meters of length spaced at 0.5 meters. Phenotyping data were collected for maturity, plant height, seed weight, and yield.

ANOVA for each trait was conducted using PROC MIXED of SAS® v. 9.4. The statistical model for the analysis was the following

$$Y_{ij}=\mu +Gi+T_{j}+\varepsilon_{ij}\;with\;i=1,2,\ldots ,250\;and\;j=1,2,3$$

Y_ij_ was the response from the i^th^ genotype in the j^th^ year, μ was the overall mean, G_i_ represented the effect of the i^th^ genotype (fixed effect), T_j_ was the effect of the j^th^ year (fixed effect), and ε_ij_ was the experimental error associated with the ij^th^ observation.

### Genotyping

DNA was extracted from young soybean trifoliate using the CTAB (hexadecyltrimethyl ammonium bromide) method [23]. DNA library was prepared using the restriction enzyme ApeKI following the GBS protocol described by Elshire et al. [24] and DNA sequencing was performed using GBS method [24, 25]. The 90-bp, pair-end sequencing was performed on each soybean genotype using the GBS protocol by an Illumina HiSeq at the Institute of Genetics and Developmental Biology, Chinese Academy of Sciences, Beijing, China. The GBS dataset contained 3.26 M short-reads or 283.74 Mbp of sequence for each accession. The short reads were aligned to the soybean whole genome sequence (Wm82.a1.v1) (https://www.soybase.org/GlycineBlastPages/archives/Gma1.01.20140304.fasta.zip; https://www.soybase.org/GlycineBlastPages/index.php?db_select=Gma1.01) using SOAPaligner/soap2 (http://soap.genomics.org.cn/) and SOAPsnp v. 1.05 was used for SNP calling [26, 27]. Approximately half a million SNPs were identified by SNP calling. The minor allele frequency (MAF) threshold less than 5%, and SNPs with heterozygosity more than 10% and or over 15% missing data were eliminated. A total of 10,259 high-quality SNPs were retained and used for further analysis.

### Population structure analysis

Population structure was inferred using STRUCTURE 2.3.4 through a Bayesian resampling technique [28]. Out of the 10,259 filtered SNPs, a total of 5,129 SNPs were randomly chosen for inferring population structure (*K*). This approach is suitable when the complete set of SNPs is computationally heavy as described by Huang et al. [29]. The analysis was run using an admixture model along with a correlated allele frequency model, which was independent of each run [30].

A total of ten runs were performed for each estimated *K*. The Markov chain Monte Carlo (MCMC) length of the burn-in period was 30,000 and the number of MCMC iterations was 50,000. The identification of optimal K was performed using STRUCTURE Harvester (http://taylor0.biology.ucla.edu/structureHarvester/) and based on the equation established by Evanno et al. [31]. The inferred population structure *K* was used to generate the structure *Q*-matrix consisting of *K* vectors. Each soybean accession was assigned to a Q cluster using a cut-off probability of 0.55. Any soybean accessions that could not be grouped in any of the clusters would be considered as admixture. Population structure was visualized using STRUCTURE PLOT with the option “sort by Q” [32].

### Genome-wide association study (GWAS)

GWAS was conducted using a Bayesian Information and Linkage Disequilibrium Iteratively Nested Keyway (BLINK) and run in R using the package ‘BLINK’ [29]. Significant SNPs were those with an LOD value greater than 3 [33].

BLINK was an improved model version of Fixed and Random Model Circulating Probability Unification (FarmCPU) and is statistically powerful and efficient in identifying significant SNPs associated with a trait of importance [29]. FarmCPU involved a fixed-effect model (FEM) and a random-effect model (REM), which were run iteratively. FarmCPU assumed an even distribution of markers within the genome. However, this assumption was relaxed in BLINK where a Linkage Disequilibrium information was used instead [29]. The REM part was replaced by a second FEM in BLINK. The two FEM models used in BLINK were the following.

$$FEM\;(1):\;y_{i}=M_{i1}b_{1}+M_{i2}b_{2}+\ldots +M_{ik}b_{k}+M_{ij}d_{j}+e_{i}$$

$$FEM\;(2):\;y_{i}=M_{i1}b_{1}+M_{i2}b_{2}+\ldots +M_{ij}b_{j}+e_{i}$$

where y_i_ was the phenotypic data from the i^th^ sample; M_i1_, M_i2_b_2_, …, M_ik_ were the genotypes of k pseudo QTNs, which were initially empty and with effects b_1_, b_2_, …, b_k_, respectively; M_ij_ represented the j^th^ genetic marker of the i^th^ sample; and e_i_ was the residual having a distribution with mean zero and a variance σ^2^_e_.

### Candidate gene discovery

A 10 kb-genomic region spanning a significant SNP was used for candidate gene search using the *G*. *max* Williams 82.a2 reference in Soybase (https://www.soybase.org/) [34]. The SNPs associated with the combined data over three years for maturity date, plant height, seed weight, and yield were used for candidate gene discovery. Functional annotations (base on which model) of each postulated candidate gene were also investigated using Soybase (https://www.soybase.org/).

### Genomic-selection and cross-validation

GS was conducted using a ridge regression best linear unbiased predictor (rrBLUP) model, which is effective in estimating the effects of loci controlling complex traits [35]. The rrBLUP model was y = WGβ + ε [17]. In this equation, y represented the vector phenotypic data; β denoted the marker effect with β~*N*(0,Iσ^2^_β_); W represented the incidence matrix relating the genotype to the vector phenotype; G was the matrix displaying the genetic marker; and ε referred to the random error. The solution for rrBLUP was β_hat = (Z^T^Z + Iλ)^-1^Z^T^y with Z = WG. The ridge parameter was defined as λ = σ^2^_e_/σ^2^_β_, where σ^2^_e_ was the residual variance and σ^2^_β_ the marker effect variance. rrBLUP was carried out in R using the ‘rrBLUP’ package [36].

Cross-validation was conducted using 4 different approaches. The first approach consisted of sampling accessions from and cross-validating within the 250 soybean genotypes (whole panel), the genotypes belonging to subpopulation 1 (Q1) from structure analysis (Q1 panel), and the genotypes from subpopulation 2 (Q2) from structure analysis (Q2 panel), respectively. For each defined subgroup, the training and validation datasets were from the same year. In the second approach the training dataset from a year was used to predict the genotype’s performance in the succeeding year(s). In the third strategy, samples from the Q1 panel was used to predict the Q2 samples’ performance within the same year and vice versa. In the fourth strategy, training the GS model with a dataset from the Q1 panel from a particular year to predict the traits of the Q2 panel from another year and vice versa. Due to the relatively small sample size of each panel, 5-fold cross-validation was carried out for GS involving the whole panel and the Q1 panel, and 3-fold cross-validation was performed for GS using the Q2 panel. Doing so allowed for an accurate estimation of the Person’s correlation coefficient (GS accuracy) that was established based on a sample size (>30) that could be statistically valid under such constraint.

ANOVA was conducted to assess the interaction effect of year and population type (whole panel, Q1 panel, and Q2 panel) on GS accuracy using PROC MIXED of SAS® v. 9.4. The statistical model for this analysis was:

$$Y_{ijk}=\mu +T_{i}+P_{j}++YP_{ij}+\varepsilon_{ijk}\;with\;i=1,2,3,j=1,2,3,\;and\;k=1,2,3,\ldots ,100$$

Y_ijk_ was the GS accuracy from the i^th^ year using the j^th^ population type at the k^th^ replication, T_i_ represented the effect of the i^th^ year (fixed effect), P_j_ was the effect of the j^th^ population type (fixed effect), and ε_ijk_ was the experimental error associated with the ijk^th^ observation.

## Results

### Phenotypic variation and correlation within traits

Data on yield and related trait were collected over 3 years on a total of 250 soybean genotypes. The broad-sense heritability (*H*^2^) of maturity, plant height, seed weight and yield was 0.74, 0.89, 0.94 and 0.66, respectively. Maturity ranged from 77.3 to 139.0 days, and plant height ranged from 18.5 cm to 162.9 cm over 3 years (S2 Table) (S1 Fig). Seed weight ranged from 3.13 (g/100 seeds) to 33.82 (g/100 seeds), and values for yield ranged from 649.4 to 4286.6 kg/hm^2^ over 3 years (S2 Table) (S2 Fig). Significant differences were identified between years and genotypes for all traits (S3 Table).

To assess consistency across years, Pearson’s correlation coefficients were also calculated for each trait across years. The correlation between years was high for maturity, seed weight, and plant height, and relatively moderate for yield. Among them, the high correlation was found for maturity (*r* = 0.763) between 2008 and 2010, seed weight (*r* = 0.876) between 2008 and 2010; Yield (*r* = 0.604) between 2008 and 2009, plant height (*r* = 0.848) between 2009 and 2010 (S4 Table).

Pearson’s correlation coefficients (r) between maturity, plant height, seed weight, and yield were computed. A significant positive correlation was found between plant height and maturity (*r* = 0.439), and a negative correlation between plant height and Seed_weight (*r* = -0.257). There were almost no correlation between maturity and seed weight, maturity and yield, seed weight and yield, plant height and yield (S4 Table).

### Population structure and genetic diversity analysis

SNP filtering resulted in 10,259 high-quality SNPs that were used for GWAS and GS. SNP number per chromosome varied from 292 (chromosome 12) to 785 (chromosome 18), with an average SNP number of 513 per chromosome (S5 Table). The average minor allele frequency (%) per chromosome ranged between 16.24% and 24.73%, with an average of 21.02% (S4 Table). The average percentage of heterozygous SNPs per chromosome varied from 0.42% to 0.74%, with an average of 0.59%. There was no significant discrepancy in the average percentage of missing SNPs per chromosome (S5 Table).

STRUCTURE Harvester indicated a delta K peak at K equal to 2 (S3A Fig), indicating that the panel involving the 250 soybean genotypes consisted of two subpopulations. The bar plot from STRUCTURE 2.3.4 showed the two-well differentiated subpopulations with an extremely low level of admixture (S3B Fig). The mean inbreeding coefficients of the subpopulation relative to the total population were 0.565 and 0.043 for the subpopulation 1 and subpopulation 2, respectively (S6 Table). The average distances between individuals in the same cluster were 0.332 (subpopulation 1) and 0.154 (subpopulation 2) (S6 Table). The overall proportions of membership of a genotype within each cluster were 0.567 and 0.433 for subpopulation 1 and subpopulation 2, respectively (S6 Table). The average allele frequency divergence among populations was 0.095 (S6 Table).

Genetic diversity analysis was also carried out along with population structure as shown in S3C Fig. A good correlation was found between the genetic diversity analysis and the structure analysis. The admixture genotypes were randomly scattered in the phylogenetic tree.

### Genome-wide association study (GWAS)

**Fig 1:**
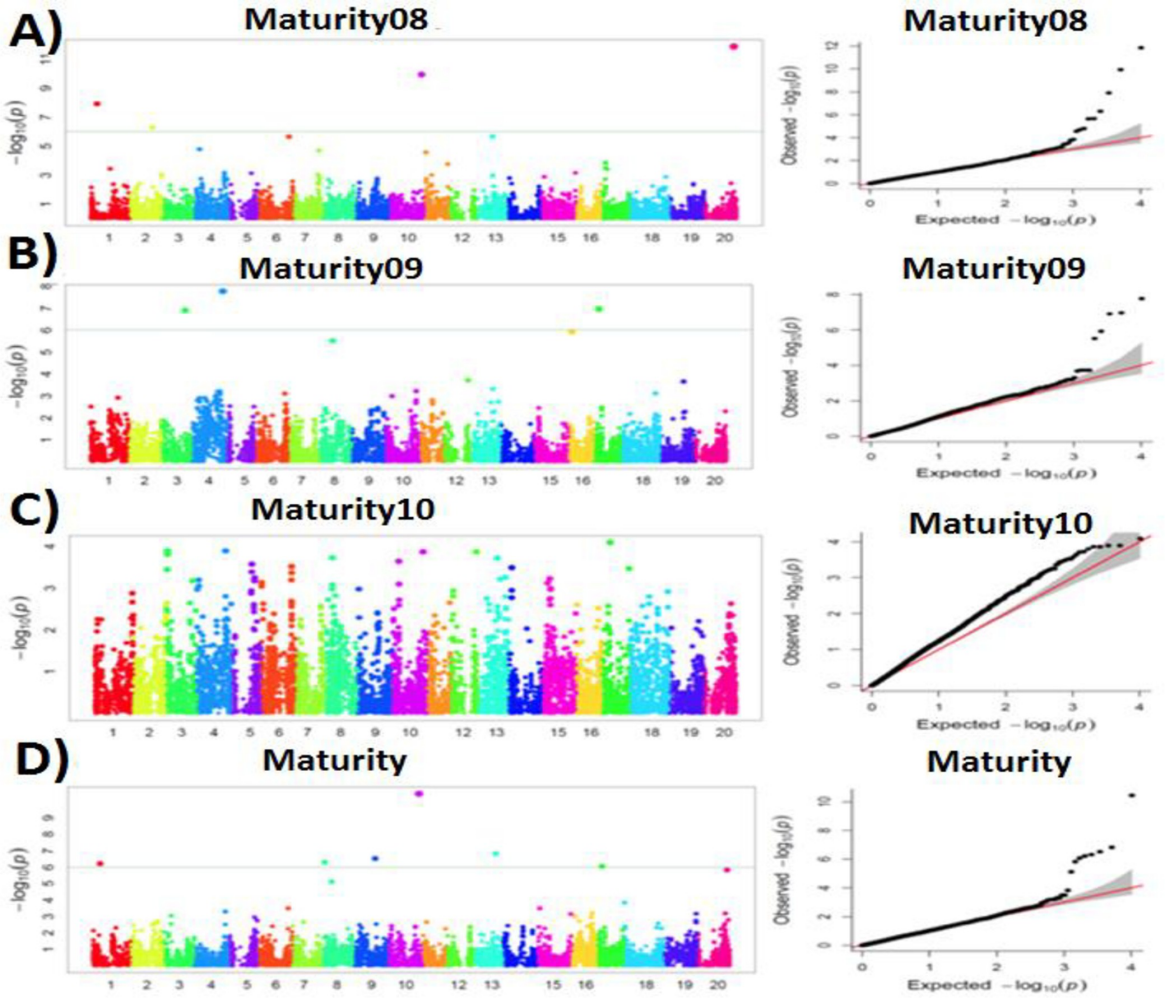
Manhattan plots and QQ-plots for maturity in 2008 (A), 2009 (B), 2010 (C), and the average data over 3 years (D).

A total of 20 SNPs associated with maturity were identified (Table 2). These SNPs were distributed across the soybean genome (Fig 1) with chromosome 20 having the most significant SNPs. The top five SNPs with the highest LOD values were located on chromosomes 10, 13, 9, 8, and 1 (Table 2). The *t-test* of the difference between genotypic class from the aforementioned SNPs was significant except for Chr13_33362588 (S4A–S4E Fig) (Table 2). A total of 31 SNPs associated with plant height were identified (Table 1). The top five SNPs were found on chromosomes 5, 19, and 20 (S6 Fig) (Table 1). The *t-test* of the data from the two genotypic classes defined by the aforementioned SNPs was significant (S4F–S4J Fig).

**Table 1:** Significant SNPs associated with the average maturity and plant height over 3 years, chromosome and physical position of the significant SNPs, LOD (-log10(p-value)) values, minor allele frequency at the SNP locus, and gene ID and functional annotation.

Traits	SNP	Chromosome	Position	LOD(-log_10_(p-value))	MAF	GeneID	Functional_annotation
Maturity	Chr01_10725106	1	10725106	6.22	14.04	NA	NA
	Chr03_10846056	3	10846056	3.06	7.48	NA	NA
	Chr04_46043483	4	46043483	3.31	6.75	NA	NA
	Chr04_46043518	4	46043518	3.31	6.75	NA	NA
	Chr06_45233584	6	45233584	3.50	13.03	*Glyma*.*06g265000*	LEUCINE-RICH REPEAT-CONTAINING PROTEIN
	Chr08_3672982	8	3672982	6.32	22.53	*Glyma*.*08g046800*	TWO-COMPONENT SENSOR HISTIDINE KINASE
	Chr08_14052904	8	14052904	5.12	16.54	*Glyma*.*08g176000*	REPLICATION PROTEIN A-RELATED
	Chr09_30962080	9	30962080	6.53	16.60	NA	NA
	Chr10_45903960	10	45903960	10.47	38.31	*Glyma*.*10g228900*	LEUCINE-RICH REPEAT RECEPTOR-LIKE PROTEIN KINASE
	Chr13_33362588	13	33362588	6.83	6.61	*Glyma*.*13g220200*	F-BOX/LEUCINE RICH REPEAT PROTEIN
	Chr15_5091735	15	5091735	3.50	6.02	*Glyma*.*15g066800*	MYB-LIKE DNA-BINDING PROTEIN MYB
	Chr15_51032135	15	51032135	3.15	22.89	*Glyma*.*15g272900*	POLY-A BINDING PROTEIN 2
	Chr16_5911007	16	5911007	3.02	7.69	*Glyma*.*16g060400*	26S PROTEASOME REGULATORY COMPLEX, SUBUNIT PSMD10
	Chr16_31347650	16	31347650	3.21	40.40	*Glyma*.*16g152800*	PPR REPEAT
	Chr17_7918542	17	7918542	6.07	13.01	*Glyma*.*17g100600*	MALATE AND LACTATE DEHYDROGENASE
	Chr18_14705	18	14705	3.84	5.10	*Glyma*.*18g000100*	RNA POLYMERASE II TRANSCRIPTION MEDIATORS
	Chr19_48601588	19	48601588	3.17	20.24	*Glyma*.*19g236900*	MYOGENIC FACTOR
	Chr20_41339091	20	41339091	3.20	25.00	*Glyma*.*20g176100*	O-METHYLTRANSFERASE
	Chr20_41339093	20	41339093	3.20	25.00	*Glyma*.*20g176100*	O-METHYLTRANSFERASE
	Chr20_43647960	20	43647960	5.83	8.27	NA	NA
Plant_height	Chr02_35754374	2	35754374	3.13	21.69	NA	NA
	Chr02_44442246	2	44442246	3.41	43.48	*Glyma*.*02g257800*	SYNTAXIN BINDING PROTEIN 5
	Chr05_4341777	5	4341777	7.97	18.11	*Glyma*.*05g048800*	CLEAVAGE AND POLYADENYLATION SPECIFICITY FACTOR
	Chr05_27107594	5	27107594	4.99	6.53	*Glyma*.*05g102100*	ARABINOGALACTAN PROTEIN 18
	Chr05_33965825	5	33965825	4.24	15.26	*Glyma*.*05g145900*	PROTEIN OF UNKNOWN FUNCTION
	Chr06_11029800	6	11029800	5.42	46.43	*Glyma*.*06g134200*	SERINE/THREONINE-PROTEIN KINASE
	Chr07_7159615	7	7159615	3.05	6.80	*Glyma*.*07g078600*	AP2 DOMAIN
	Chr08_8813970	8	8813970	3.78	6.67	*Glyma*.*08g115100*	ALPHA TUBULIN
	Chr08_9862761	8	9862761	3.12	21.19	*Glyma*.*08g127900*	KELCH REPEAT DOMAIN
	Chr08_17577833	8	17577833	3.58	10.92	NA	NA
	Chr10_5011226	10	5011226	3.06	27.50	*Glyma*.*10g055000*	ABC TRANSPORTER
	Chr10_6919622	10	6919622	4.96	9.27	NA	NA
	Chr11_5824301	11	5824301	3.02	32.16	*Glyma*.*11g077500*	BETA CATENIN-RELATED ARMADILLO REPEAT-CONTAINING
	Chr11_7484586	11	7484586	3.58	23.62	*Glyma*.*11g098500*	POLLEN PROTEINS OLE E I LIKE
	Chr11_33650868	11	33650868	3.31	12.55	*Glyma*.*11g242300*	ATP-DEPENDENT RNA HELICASE
	Chr11_34656506	11	34656506	3.30	10.12	*Glyma*.*11g256800*	RNA POLYMERASE II TRANSCRIPTION MEDIATORS
	Chr13_23110902	13	23110902	3.84	8.05	*Glyma*.*13g118200*	SWI/SNF-RELATED MATRIX-ASSOCIATED ACTIN-DEPENDENT REGULATOR OF CHROMATIN SUBFAMILY-RELATED
	Chr13_42643620	13	42643620	3.01	47.23	*Glyma*.*13g331800*	EXOCYST COMPLEX COMPONENT 7
	Chr16_2501308	16	2501308	3.15	5.98	*Glyma*.*16g025800*	TOPOISOMERASE-RELATED PROTEIN
	Chr18_280602	18	280602	3.33	28.74	*Glyma*.*18g003300*	PENTATRICOPEPTIDE REPEAT (PPR) SUPERFAMILY PROTEIN
	Chr19_44603046	19	44603046	6.21	10.36	*Glyma*.*19g187900*	PHD-FINGER
	Chr19_45270675	19	45270675	6.60	14.06	*Glyma*.*19g195500*	UBIQUITIN
	Chr19_45322411	19	45322411	3.77	25.61	*Glyma*.*19g196000*	TETRATRICOPEPTIDE REPEAT PROTEIN, TPR
	Chr19_45326559	19	45326559	4.06	25.59	*Glyma*.*19g196000*	TETRATRICOPEPTIDE REPEAT PROTEIN, TPR
	Chr19_45326600	19	45326600	4.38	25.50	*Glyma*.*19g196000*	TETRATRICOPEPTIDE REPEAT PROTEIN, TPR
	Chr19_45359939	19	45359939	4.04	20.24	NA	NA
	Chr19_45769759	19	45769759	11.78	23.53	*Glyma*.*19g200800*	TRANSCRIPTION FACTOR NF-Y ALPHA-RELATED
	Chr20_423349	20	423349	3.61	13.79	NA	NA
	Chr20_442209	20	442209	3.25	12.35	*Glyma*.*20g004400*	ZINC FINGER DHHC DOMAIN CONTAINING PROTEIN
	Chr20_34864638	20	34864638	3.76	21.37	*Glyma*.*20g106200*	AMINO ACID TRANSPORTER
	Chr20_43000412	20	43000412	6.76	43.83	NA	NA

A total of 37 SNPs were found to be associated with seed weight (Table 2). The top five significant SNPs were located on chromosomes 4, 9, 1, 17, and 8, respectively (S7 Fig) (Table 2). *T-test* analysis was significant between genotypic class for these SNPs (S4K–S4O Fig). Of the 37 SNPs associated with the average seed weight over three years, 10 were located on chromosome 10 (Table 2). A total of 23 significant SNPs were identified for yield (Table 1) (S5D Fig). The five most significant SNPs were located on chromosomes 9, 19, 8, 10, and 7, respectively (Table 2). The variation between each genotypic class defined by the aforementioned SNPs was visualized in S5P–S5T Fig. Of the 23 significant SNPs, 6 were located on chromosome 10 (Table 2). Of the 6 significant SNPs found on chromosome 10, 3 were mapped within a 3-Mb genomic region (Table 2), indicating a strong likelihood of QTL(s) affecting soybean yield in this region.

**Table 2:** Significant SNPs associated with the average seed-weight and yield over 3 years, chromosome and physical position of the significant SNPs, LOD (-log10(p-value)) values, minor allele frequency at the SNP locus, and gene ID and functional annotation.

Traits	SNP	Chromosome	Position	LOD(-log^10^(p-value))	MAF	GeneID	Functional_annotation
Seed weight	Chr01_7408096	1	7408096	4.77	13.36	NA	NA
	Chr01_42908212	1	42908212	10.47	5.14	*LOC100801248*	PROTEIN TIC 56, CHLOROPLASTIC-LIKE
	Chr02_695027	2	695027	3.63	7.53	*Glyma*.*02g006500*	CHAPERONE-ACTIVITY OF BC1 COMPLEX (CABC1)-RELATED
	Chr02_19239630	2	19239630	6.60	5.98	*Glyma*.*02g161100*	CAMP-RESPONSE ELEMENT BINDING PROTEIN-RELATED
	Chr04_36949349	4	36949349	22.58	16.47	NA	NA
	Chr05_34421673	5	34421673	3.65	20.00	*Glyma*.*05g150300*	TIC22-LIKE FAMILY
	Chr07_33588669	7	33588669	7.32	9.58	NA	NA
	Chr08_5242876	8	5242876	4.08	35.63	*Glyma*.*08g068300*	DNAJ DOMAIN
	Chr08_11477277	8	11477277	3.47	43.90	*Glyma*.*08g149600*	FALZ-RELATED BROMODOMAIN-CONTAINING PROTEINS
	Chr08_11495775	8	11495775	3.81	42.92	*Glyma*.*08g149700*	UNCHARACTERIZED CONSERVED PROTEIN
	Chr08_47483065	8	47483065	7.85	25.83	*Glyma*.*08g363300*	NA
	Chr09_42124679	9	42124679	12.97	35.90	*Glyma*.*09g196700*	RING FINGER DOMAIN-CONTAINING
	Chr09_42432921	9	42432921	6.71	42.29	*Glyma*.*09g199800*	UNCHARACTERIZED
	Chr10_7815951	10	7815951	3.14	10.59	*Glyma*.*10g075300*	TETRATRICOPEPTIDE REPEAT PROTEIN, TPR
	Chr10_12259917	10	12259917	3.52	7.69	*Glyma*.*10g091000*	ALPHA-N-ACETYLGLUCOSAMINIDASE
	Chr10_16854456	10	16854456	3.01	11.51	NA	NA
	Chr10_18370776	10	18370776	3.40	13.65	*LOC102668189*	SERINE/THREONINE-PROTEIN PHOSPHATASE 7 LONG FORM
	Chr10_19170955	10	19170955	3.03	12.55	NA	NA
	Chr10_19620114	10	19620114	3.32	11.93	NA	NA
	Chr10_20805615	10	20805615	3.32	12.50	NA	NA
	Chr10_24454215	10	24454215	3.10	15.09	NA	NA
	Chr10_24773660	10	24773660	3.57	14.29	NA	NA
	Chr10_29894008	10	29894008	7.84	24.05	NA	NA
	Chr11_17752345	11	17752345	6.60	11.81	*LOC100787408*	NA
	Chr12_611590	12	611590	3.04	9.47	*Glyma*.*12g008200*	PUTATIVE TRANSMEMBRANE PROTEIN CMP44E
	Chr13_21793355	13	21793355	4.40	10.20	*Glyma*.*13g102900*	GDSL/SGNH-LIKE ACYL-ESTERASE
	Chr13_26873585	13	26873585	3.03	31.10	NA	NA
	Chr14_48362592	14	48362592	6.31	9.84	NA	NA
	Chr15_27399758	15	27399758	4.58	14.47	*LOC106796140*	NA
	Chr15_33423211	15	33423211	3.32	25.82	*Glyma*.*15g212800*	DEDICATOR OF CYTOKINESIS (DOCK)
	Chr17_7302504	17	7302504	5.38	7.05	*LOC100795267*	NA
	Chr17_15088391	17	15088391	9.68	23.08	NA	NA
	Chr18_51853903	18	51853903	3.31	8.10	*Glyma*.*18g229500*	PPR REPEAT
	Chr18_51854710	18	51854710	3.78	7.72	*Glyma*.*18g229500*	PPR REPEAT
	Chr18_53835731	18	53835731	4.73	14.96	*Glyma*.*18g251800*	PUTATIVE SERINE/THREONINE PROTEIN KINASE
	Chr20_41923741	20	41923741	6.68	12.35	*Glyma*.*20g181100*	LEUCINE-RICH REPEAT RECEPTOR-LIKE PROTEIN KINASE
	Chr20_46703305	20	46703305	3.42	6.40	*Glyma*.*20g234500*	MYB-LIKE DNA-BINDING DOMAIN
Yield	Chr01_28292204	1	28292204	5.32	7.26	*Glyma*.*01g093300*	RNA RECOGNITION MOTIF
	Chr02_12086588	2	12086588	3.96	40.00	*Glyma*.*02g121600*	MADS BOX PROTEIN
	Chr03_42920496	3	42920496	3.12	44.44	*Glyma*.*03g227300*	PHYTOCHROME REGION
	Chr07_7610107	7	7610107	6.69	49.17	*Glyma*.*07g082800*	HOMO-OLIGOMERIC FLAVIN CONTAINING CYS DECARBOXYLASE FAMILY
	Chr07_38409249	7	38409249	3.58	7.76	*Glyma*.*07g212500*	LONGEVITY ASSURANCE FACTOR 1 (LAG1)
	Chr07_38409765	7	38409765	3.52	9.96	*Glyma*.*07g212500*	LONGEVITY ASSURANCE FACTOR 1 (LAG1)
	Chr08_47747059	8	47747059	8.43	41.43	*Glyma*.*08g366900*	ZINC FINGER PROTEIN WITH KRAB AND SCAN DOMAINS
	Chr09_3204462	9	3204462	8.92	25.98	*Glyma*.*09g038300*	CALMODULIN-BINDING TRANSCRIPTION ACTIVATOR (CAMTA)
	Chr10_2089552	10	2089552	7.02	24.31	*Glyma*.*10g023900*	UNCHARACTERIZED CONSERVED PROTEIN
	Chr10_19477000	10	19477000	3.29	31.62	NA	NA
	Chr10_24773517	10	24773517	3.42	29.51	NA	NA
	Chr10_26343503	10	26343503	3.63	22.55	NA	NA
	Chr10_27017034	10	27017034	4.33	24.26	NA	NA
	Chr10_29778879	10	29778879	5.40	23.23	NA	NA
	Chr13_29543721	13	29543721	4.87	12.30	NA	NA
	Chr16_30502283	16	30502283	5.10	45.90	*Glyma*.*16g144600*	GTP-BINDING PROTEIN ALPHA SUBUNIT
	Chr17_32451159	17	32451159	4.76	40.16	*Glyma*.*17g203300*	O-FUCOSYLTRANSFERASE FAMILY PROTEIN
	Chr18_1543178	18	1543178	5.84	5.69	*Glyma*.*18g021100*	GAMMA-GLUTAMYLTRANSFERASE
	Chr18_2896796	18	2896796	4.69	32.11	*Glyma*.*18g037000*	RING ZINC FINGER PROTEIN
	Chr18_9093423	18	9093423	6.24	24.57	NA	NA
	Chr19_9586720	19	9586720	8.52	27.97	NA	NA
	Chr19_44770496	19	44770496	3.72	28.80	*Glyma*.*19g190000*	TRANSCRIPTION FACTOR GT-2 AND RELATED PROTEINS
	Chr19_48289439	19	48289439	3.67	30.99	*Glyma*.*19g232800*	RING FINGER DOMAIN-CONTAINING

### Candidate genes selection

Candidate genes found within a 10-kb genomic region harboring a significant SNP associated for maturity, plant height, seed weight, and yield were investigated. For maturity, 20 significant SNPs were identified (Table 1). A total of 14 were mapped to genomic regions harboring annotated genes in Soybase (www.soybase.org). The annotated genes had a wide variety of functions. Leucine-rich repeat (LRR) domain was prevalent as shown in Table 1. Of the 31 significant SNPs associated with plant height, 25 were found in the vicinity of annotated genes (Table 1). The candidate genes found near the most significant SNPs were *Glyma*.*19g200800*, *Glyma*.*05g048800*, *Glyma*.*19g195500*, *Glyma*.*19g187900*, and *Glyma*.*06g134200*, encoding transcription factor NF-Y alpha-related, cleavage and polyadenylation specificity factor, ubiquitin, PHD-finger, and serine/threonine-protein kinase, respectively (Table 1).

A total of 24 candidate genes were found for seed weight. Of which, 19 had functional annotations and one has an uncharacterized function (Table 2). GWAS for seed weight revealed six QTL(s) on chromosome 10; however, most of the SNPs found on the chromosome 10 were not mapped in the vicinity of an annotated gene (Table 2). Functional annotations related to the candidate genes had diverse functions. A total of 15 annotated genes were found near the significant SNPs associated with yield, of which, 14 had functional annotations. Similar to what was found for seed weight, only one candidate gene was identified on chromosome 10 which harbored 10 SNPs associated with yield (Table 2). Most of the candidate genes encoded transpiration factors and transferases.

### Genomic selection (GS) in yield and related traits

**Fig 2:**
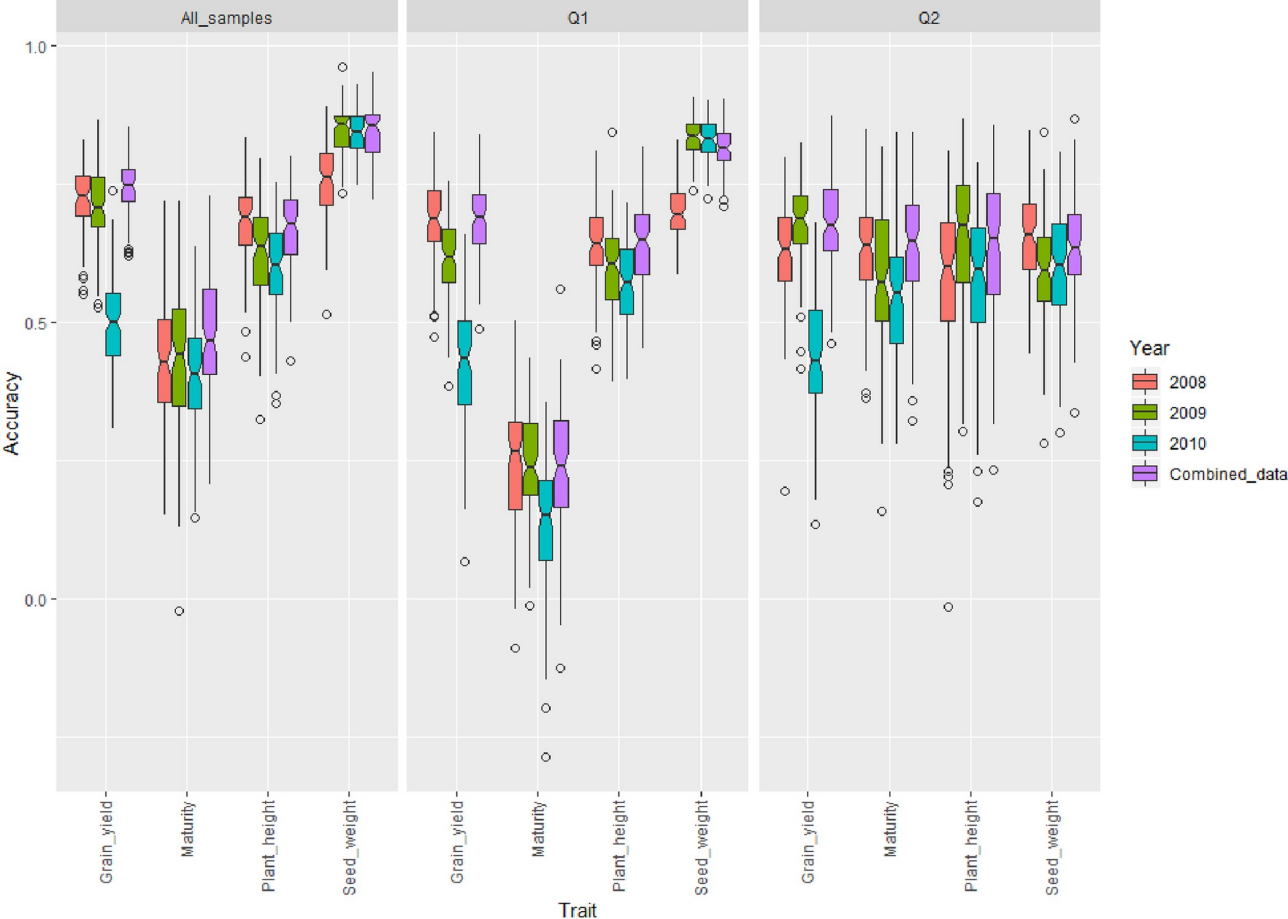
Genomic selection accuracy for yield, maturity, plant height, and seed weight using training/testing sets from all 250 soybean accessions (all samples), samples derived from Q1, and samples from the Q2 subpopulation. Cross-validation was conducted using the data from the same year.

Prediction accuracy for yield and yield-related traits were calculated by using a 5-fold cross-validation within the 250-soybean accession panel (Fig 2) (S7 Table). The between-year variation was relevant for yield where the highest prediction accuracy was 0.72 in 2008 and the lowest one was 0.50 in 2010 (Fig 2) (S7 Table). The prediction accuracy was also calculated using the two subpopulations derived from structure analysis. Cross-validation using all 250 soybean accessions and samples from the Q1 group provided a similar trend as shown in Fig 2, Compared to the whole panel, prediction accuracy for maturity was reduced in Q1 panel but increased in Q2 panel (Fig 2). On average, the prediction accuracy was the highest for seed weight (0.84) and was the lowest for maturity (0.47) (S7 Table). Interestingly, prediction accuracy averaged 0.64 with a less variation between traits and across years when samples from Q2 were used to estimate prediction accuracy of seed weight (Fig 2). In addition, accuracy for predicting maturity was the best using Q2 samples (Fig 2) (S7 Table). The ANOVA results indicated a statistically significant interaction effect between the year and population type on the prediction accuracy of GS for maturity, plant height, seed weight, and yield (S8 Table). The results indicated that year and population structure were important factors to take into account when evaluating the prediction accuracy in the GS approach.

**Fig 3:**
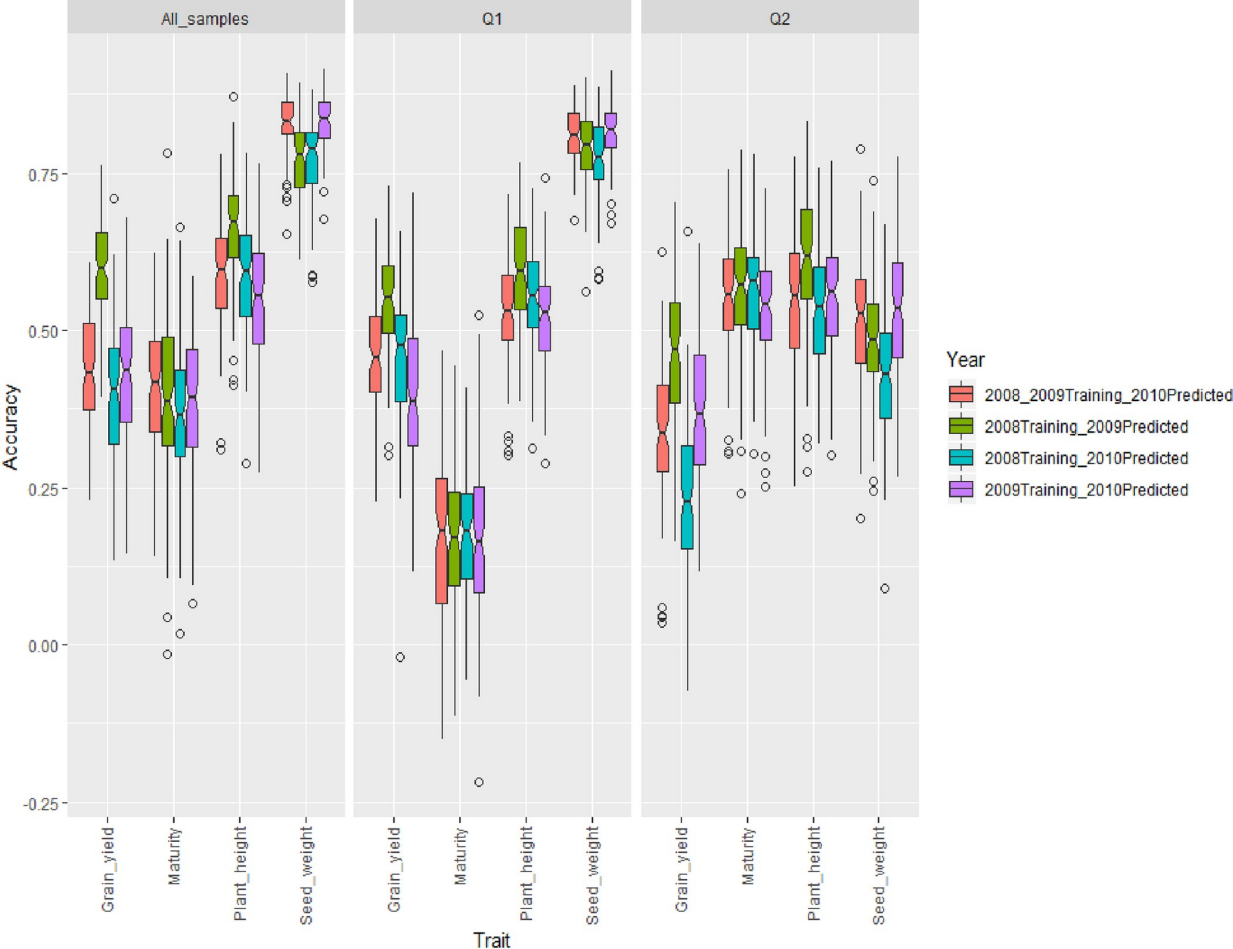
Genomic selection accuracy for yield, maturity, plant height, and seed weight using training/testing sets from all 250 soybean accessions (all samples), samples derived from Q1, and samples from the Q2 subpopulation. Cross-validation was conducted in a way that the data from a year was used to predict that of from the succeeding year(s).

Prediction accuracy using datasets from different years had similar trends to within-year cross validation (Figs 2 and 3). Overall, there is a lack of consistency between prediction accuracy and the training year across traits and population types. This could be explained by the significant interaction effect of population structure and years on prediction accuracy of plant height, seed weight, and yield (S9 Table). Yield in 2010 was better predicted using dataset from 2009 than using yield data obtained in 2008 when all samples within the panel and individuals from Q2 were used for cross-validation, respectively (Fig 3) (S10 Table). However, plant height in 2010 was better predicted by the dataset recorded in 2008 for all sample- and Q1 sample-cross validation. Different results were found for plant height when cross-validation was carried out using individuals from the Q2 group (Fig 3). Genomic prediction appeared to be year-independent but subpopulation-dependent for maturity (Fig 3) (S10 Table). ANOVA results showed that there was no significant interaction between population structure and year in predicting maturity (S9 Table). In addition, year effect on prediction accuracy of maturity was not significant (S9 Table). However, the prediction accuracy for maturity was heavily influenced by population structure (S9 Table). Maturity could be better predicted than the other traits when samples from the group Q2 were used for cross-validation (Fig 3) (S10 Table).

Prediction accuracy was also calculated using samples from Q1 as the training set and samples from Q2 as the testing set and vice versa. For cross-validation using data from the same year (S11 Table), a discrepancy in prediction accuracy was found when samples from one subpopulation were used to predict the ones from the other group (S8 Fig). Overall, prediction accuracy was slightly higher for most traits when the GS model was trained using samples from subpopulation 1 (S8 Fig). This difference was substantial for maturity. The average prediction accuracy for maturity was 0.49 and 0.20 for Q1-based training set and Q2-based population, respectively (S11 Table). Unlike the two previous approaches which cross-validation was carried out using within-subgroup samples, the variation of prediction accuracy between years was more pronounced when the prediction was done across subpopulations (S8 Fig). Also, the interaction effect of population structure and year on prediction accuracy was significant for maturity, plant height, seed weight and yield (S8 Table). When the training set was taken from Q1 in order to predict yield of Q2, prediction accuracy was 0.72 and 0.41 for 2009 and 2008, respectively (S11 Table). A similar trend was found for yield when the training samples were derived from Q2 to test on Q1 (S8 Fig).

In addition to performing a within-year prediction accuracy using two subpopulations, trait prediction for other years of a genetically-distant population subset was also conducted. Overall, results indicated a similar trend to what was found using a within-year prediction approach (S9 Fig). The interaction effect of population structure and year on prediction accuracy was significant for maturity, plant height, seed weight, and yield (S9 Table). Prediction accuracy for most traits was slightly higher when the samples from Q1 were used to train the prediction model (S12 Table). Training the model on Q1 resulted in prediction accuracy of yield being 0.62, 0.40, 0.36, and 0.41 when the training/testing year was 2008/2009, 2008/2010, 2009/2010, average 2008_2009/2010, respectively (S12 Table). When the prediction model was trained using samples from Q2, the prediction accuracy for yield was 0.28, 0.13, 0.31, and 0.21 corresponding to the training/testing years 2008/2009, 2008/2010, 2009/2010, average 2008_2009/2010, respectively (S12 Table). Prediction accuracies were increased on plant height, maturity date, and seed weight each year helped increase GS when the model was trained under Q2. The improvement was achieved by averaging the training set data from 2008 and 2009 to predict the testing set in 2010. Prediction accuracy was 0.47, 0.07, and 0.60 for plant height, maturity date, and seed weight, respectively, using the 2008 data from the Q2 samples to predict the 2010 data from the Q1 samples (S9 Fig) (S12 Table). By taking the combined data from 2008 and 2009 to establish the training set, plant height, maturity date, and seed weight of the Q2 samples were predicted with an accuracy of 0.52, 0.09, and 0.68, respectively (S12 Table). These results indicated that prediction accuracy was impacted by multiple factors such as population structure and the variable year from which the training set was established.

## Discussion

GWAS was conducted using a BLINK model. BLINK is one the latest and most improved statistical models to conduct GWAS [29]. Spurious associations could be reduced by incorporating population structure and Kinship effects into the GWAS model [37]. This has been established into the BLINK algorithm [29]. Therefore, we did not run the MLM (Q + K) of Tassel 5 for GWAS [38] since previous investigations had successfully demonstrated that BLINK had more statistical power in identifying true associations and reducing false positives for a large number of traits [29]. The only purpose of running population structure (Q) in this study was to assess GS accuracy between two unrelated subpopulations, which will be further discussed in this report.

A total of 20, 37, 31, and 23 SNPs were found to be significantly associated with maturity, seed weight, plant height, and yield, respectively, using the combined data obtained over 3 years (Tables 1 and 2). Diers et al. [39] also reported a similar range of SNP number associated with these traits using a nested association mapping (NAM) soybean population. A total of 19, 29, 15, and 23 SNPs were reported to be associated with maturity, seed weight, plant height, and yield, respectively [39]. Assefa et al. [40] found a total of 14, 10, and 9 SNPs associated with seed weight, plant height, and yield, respectively, based upon a GWAS study involving a total of 419 soybean accessions. After SNP validation is performed, the information from this report could be used in trait introgression efforts in soybean breeding programs. This has been successfully demonstrated by Hegstad et al. [41] who introgressed large-effect QTL regions from commercial soybean cultivars with high yield into the Corteva Agriscience soybean accessions.

Previous investigations reported a total of more than 60 loci controlling maturity in Soybase (https://www.soybase.org/). Chromosome 16 has been shown to harbor the most significant loci affecting soybean maturity. Of the 20 SNPs found to be associated with the average maturity over 3 years in this study, one was mapped at 31 Mb on chromosome 16. Diers et al. [39] found a total of 19 regions for maturity on chromosome 16. However, Wang et al. [42] reported a significant discrepancy in SNPs associated with maturity across multiple environments, which was also consistent with that of reported in this study where different SNPs were reported in different years with different environmental conditions. A cluster of significant SNPs were identified in a 232-kb region of chromosome 20. This region spanned a significant SNP associated with maturity that was reported by Zatybekov et al. [43]. A high-LOD SNP (LOD>10), Chr10_45903960, associated with maturity was also found on chromosome 10. A total of 10 loci on chromosome 10 were reported to be associated with maturity in Soybase (https://www.soybase.org/). One of those loci harbored the SNP Chr10_45903960. Two of the SNPs associated with maturity, Chr10_45903960 and Chr20_41339091, were located into the *E2* and *E4* loci that have been reported to control maturity in soybean [44]. For the plant height-related SNPs, of 31 SNPs found to be associated with the average height over 3 years in this study, a new plant height locus was mapped to chromosome 20 (Chr20_43000412, LOD = 6.76, MAF = 43.83%). Previous investigations showed discrepancy in terms of SNP location. Zatybekov et al. [43] reported two SNPs significantly associated with plant height on chromosome 9 and 20, which were mapped at 42 Mb and 8 Mb, respectively. Our results did not indicate any plant-height associated SNPs on chromosome 9, whereas the one mapped on chromosome 20 is about 7.5 Mb away from that of reported by Zatybekov et al. Of the 68 loci associated with plant height in Soybase (https://www.soybase.org/), 19 loci were found on chromosome 19. The plant height-associated SNP with the highest LOD value found in this study was mapped on chromosome 19. We found that all significant SNPs associated with plant height and mapped on chromosome 19 overlapped with the *Dt1* locus, which has been well-described for controlling plant height in soybean [7]. These findings suggest that the present investigation contributes towards enriching SNP markers associated with plant height, which is essential in efficiently establishing a breeding pipeline for agronomic trait improvement in soybean.

Zhang et al. [1] reported that seed weight was a complex trait controlled by a large number of loci. This statement appeared to be sound when taking into account the number of SNPs associated with maturity reported in this study. To date, more than 100 QTLs affecting seed weight have been reported (https://www.soybase.org/). A large number of these QTLs were mapped on chromosomes 2, 4, 5, 7, 17, 18, and 20 [39, 43]. Our results revealed a SNP with an LOD of 22.58 at 37 Mb on chromosome 4, indicating the likelihood of a strong QTL affecting seed weight in this region. In addition, a large cluster of SNPs were found on chromosome 10. Some of which overlapped with previously reported seed weight-related QTLs (https://www.soybase.org/). For yield, previous reports showed that SNP markers associated with yield were scattered across the soybean genome. To date, more than 170 loci have been associated with yield in soybean (https://www.soybase.org/). Zatybekov et al. [43] mapped SNP markers associated with soybean yield on chromosomes 14, 17, and 20. Diers et al. [39] reported 23 loci affecting soybean yield on chromosome 16 alone. Two of the SNPs on chromosome 19 reported in this study were in the vicinity of a significant yield SNP marker identified by Assefa et al. [40]. Despite the lack of overlapping SNPs between traits, overlapping significant loci were identified to control two or more traits. A 7.2-Mb region of chromosome 2 and defined by the SNP markers Chr02_12086588 and Chr02_19239630 were associated with both seed weight and plant height. A 9-Mb region of chromosome 4 harboring the SNPs Chr04_46043483, Chr04_46043518, and Chr04_36949349 was significantly associated with both maturity date and seed weight. A genomic DNA sequence spanning a 450-Kb region of chromosome 7 harbored the SNPs Chr07_33588669 and Chr07_7610107 and was associated with seed weight and yield, respectively. In addition, a 270-Kb region of chromosome 8 defined by the significant SNP markers Chr08_47483065 and Chr08_47747059 were associated with both seed weight and yield. One of the most important regions reported in this investigation is defined by an 11.5-Mb region of chromosome 10 containing the significant SNPs Chr10_18370776, Chr10_19170955, Chr10_19620114, Chr10_20805615, Chr10_24454215, Chr10_24773660, Chr10_29894008, Chr10_19477000, Chr10_24773517, Chr10_26343503, Chr10_27017034, and Chr10_29778879. These significant SNPs were associated with seed weight and yield. A 4-Mb region of chromosome of chromosome 19 was found to be associated with maturity, plant height, and yield in soybean, which was in agreement with a study investigated by Assefa et al. [40]. A 5.4-MB region of chromosome 20 harbored loci that were associated with maturity, plant height, and seed weight. These findings were consistent with previously reported studies [39, 43].

Our results suggested that maturity-associated candidate genes encoding Leucine-rich repeat proteins were prevalent. Osakabe et al. [45] showed that LRR proteins acted as a key regulator for maturity in plant. In addition, Jinn et al. [46] reported that these proteins were also involved in floral organ abscission. These previous investigations supported that the LRR domains found in this study could be good candidate genes for maturity in soybean and could be further investigated towards validation. The findings indicated O-methyltransferase being a candidate gene for maturity in soybean. Held et al. [47] found that O-methyltransferase-related genes were highly expressed during cell maturation in maize. The candidate genes associated with plant height consisted of transcription factors, kinases, and biomolecule transporters. One of these transcription factors is NF-Y alpha-related. Zhao et al. [48] reported that this transcription factor regulated plant growth, indicating that NF-Y alpha-related could be a good candidate gene for plant height in soybean. The SNP markers associated with plant height and mapped in the *Dt1* locus on chromosome 19 were in the vicinity of a gene that encodes for a tetratricopeptide repeat protein (TPR). However, the role of TPR domains in affecting plant height has been poorly investigated. The candidate genes associated with seed weight that were reported in this study had diverse functional annotations. A large number of candidate genes playing significant roles in seed development were hormone-signaling [49]. However, no candidate genes involved in hormone signaling pathways were identified. A large number of candidate genes found for yield were transcription factors and transferases. These results were consistent with that of reported by Diers et al. [39] who reported candidate transcription factors that could affect yield in soybean. Candidate genes associated with maturity, plant height, seed weight, and yield were reported in this study. Additional investigations would be required to validate these candidate genes.

Prediction accuracy for maturity, plant height, seed weight, and yield was assessed. Howard and Jarquin [50] reported that GS performed better than phenotypic selection in soybean when dealing with complex trait. In this study, we found that the trend of prediction accuracies were similar for all traits when all samples and Q1 samples, respectively, were used for cross validation (Figs 2 and 3). Interestingly, prediction accuracy for plant height was lower than yield when cross-validation was conducted using data from the same year from all samples and Q1 samples, respectively. These results were different from that of reported by Ma et al. [51] who also used a one-year data to estimate prediction accuracy for plant height and yield in soybean. They reported a prediction accuracy of 0.86 and 0.47 for plant height and yield, respectively. However, when cross-validation was conducted using data from different years regardless of the subpopulation, prediction accuracy was higher for plant height than yield. This could be explained by the fact that plant height has higher heritability than yield [51], thus resulting in a higher genomic prediction accuracy. In addition, inconsistency in results has been found in previous studies investigating prediction accuracy for yield in soybean. Jarquín et al. [21] suggested an accuracy of 0.64 for yield, whereas Stewart-Brown et al. [52] and Duhnen et al. [20] reported an accuracy of 0.26 and 0.39, respectively. We have also highlighted the effect of population structure on the prediction accuracy. The results indicated that prediction accuracy for maturity was heavily affected by population structure. This could be explained by the fact maturity can cause a structure within a population, thus using maturity data from one subpopulation to predict the maturity data from another unrelated subpopulation will decrease the prediction accuracy. We have also found that year can significantly affect prediction accuracy, implying that updating the training model each year will be necessary for efficiently establishing a GS pipeline within a breeding program for agronomic trait improvement in soybean.

## Conclusion

In this report, significant differences in plant height, maturity, seed weight, and yield were identified among the genotypes. In addition, the year effect was also significant. Molecular markers associated with the above traits were identified. GS accuracy in this study varies from low to moderate and is affected by population structure and year. The SNP markers identified in this study contributed towards enriching SNP markers associated with the above traits, which were essential in efficiently establishing a marker-assisted selection (MAS) and GS pipeline for agronomic trait improvement in soybean.

## Supporting information

S1 Fig Distribution of maturity and plant height in 2008, 2009, and 2010, and the average of the 3-year data. (PPT)

S2 Fig Distribution of seed weight and yield in 2008, 2009, and 2010, and the average of the 3-year data. (PPT)

S3 Fig Population structure and genetic diversity analysis. (A) Plot showing the delta K values on the y-axis and the corresponding K values on the x-axis. The plot was obtained from STRUCTURE Harvester (Earl and VonHoldt, 2011; http://taylor0.biology.ucla.edu/structureHarvester/). The delta K peak corresponds to K = 2. (B) Bar plot showing the population structure using STRUCTURE 2.3.4 (Pritchard et al. 2000) where the red color corresponds to cluster 1 and the green one to cluster 2. The y-axis of the bar plot indicates the proportion of membership of a genotype to each cluster. (C) Phylogenetic tree involves a combined analysis between population structure and genetic diversity. The solid red circles is for subpopulation 1 and the solid green circles is for subpopulation 2, solid blue circles is for admixture.
(PPT)

S4 Fig Manhattan plots and QQ-plots for yield in 2008 (A), 2009 (B), 2010 (C), and the combined data over 3 years (D).
(PPT)

S5 Fig Boxplots showing the variation in plant maturity (A, B, C, D, and E), plant height (F, G, H, I, and J), 100-seed weight (K, L, M, N, and O), and grain yield (P, Q, R, S and T) within each genotypic class defined by the top 5 significant SNPs for each trait. The x-axis showed the genotypic class from each SNP, the y-axis showed the phenotypic value for each trait. On the y-axis, mat_snp, height_snp, seed_snp, and yield_snp denotes the maturity date, plant height, 100-seed weight and grain yield, respectively.
(PPT)

S6 Fig Manhattan plots and QQ-plots for plant height in 2008 (A), 2009 (B), 2010 (C), and the combined data over 3 years (D).
(PPT)

S7 Fig Manhattan plots and QQ-plots for seed weight in 2008 (A), 2009 (B), 2010 (C), and the combined data over 3 years (D).
(PPT)

S8 Fig Genomic selection accuracy for plant height, maturity, seed weight and yield using samples from Q1 as a training set and individuals from Q2 as a testing set, and vice versa. Cross-validation was done using data from the same year.
(PPT)

S9 Fig Genomic selection accuracy for plant height, maturity, seed weight and yield using samples from Q1 as a training set and individuals from Q2 as a testing set, and vice versa. Cross-validation was performed using the data from a year to predict that of from the succeeding year(s).
(PPT)

S1 Table List of genotypes evaluated for maturity, plant height, seed weight, and yield over three years. SD represents the standard deviation for each trait over three years.
(XLSX)

S2 Table Descriptive statistics for maturity (days), plant height (cm), seed weight (g/100 seeds) and yield (kg/hm2) among 250 soybean accessions over three years. (XLSX)

S3 Table ANOVA for maturity (days), plant height (cm), seed weight (g/100 seeds), and yield (kg/hm2). (XLSX)

S4 Table Pearson’s correlation coefficients (r) between maturity, seed weight, yield, and plant height, and between years for each trait. (XLSX)

S5 Table Distribution of a total of 10259 high-quality SNPs among the 20 haploid chromosomes, average distance between two adjacent SNPs, minor allele frequency (MAF), average percentage of heterozygote SNP, and average percentage of missing SNP per chromosome. (XLSX)

S6 Table Mean Fst values, average distance between samples within the same subpopulation, average probability value of from each individual within each cluster, and allele frequency divergence among populations. (XLSX)

S7 Table Genomic selection accuracy for maturity, plant height, seed weight and yield using 100 replications and where cross-validation was performed within all samples, samples from subpopulation Q1, and samples from subpopulation Q2, respectively. (XLSX)

S8 Table ANOVA table relating the effect of population structure, year, and interaction effect between population structure and year on the genomic prediction of maturity (days), plant height (cm), seed weight (g/100 seeds), and yield (kg/hm2). (XLSX)

S9 Table ANOVA table showing the effect of population structure, years from which the training and testing sets were established, respectively, and interaction effect between population structure and year on the genomic prediction of maturity (days), plant height (cm), seed weight (g/100 seeds), and yield (kg/hm2). (XLSX)

S10 Table Genomic selection accuracy where cross-validation was performed within all samples, samples from subpopulation Q1, and samples from subpopulation Q2, respectively. Cross-validation within each group was conducted as following. The data from 2008 were used to predict the data from 2009 and 2010, respectively, the data from 2009 were used to predict the data from 2010, and the average data from 2008 and 2009 were used to predict the data from 2010.
(XLSX)

S11 Table Genomic selection accuracy for maturity, plant height, seed weight, and yield using samples from subpopulation 1 (Q1) as training set and samples from subpopulation 2 (Q2) as testing set and vice versa. Estimation of genomic selection accuracy was done using 100 replications.
(XLSX)

S12 Table Genomic selection for maturity, plant height, 100-seed weight, and grain yield using samples from subpopulation 1 (Q1) as training set and samples from subpopulation 2 (Q2) as testing set, and vice versa. Data from 2008 in the training set were used to predict that of 2009 and 2010 in the testing set, respectively. Data from 2009 in the training set were used to predict that of 2010 in the testing set. The average data from 2008 and 2009 in the training set were used to predict that of 2010 in the testing set.
(XLSX)
