# Supplementary figures and images for: Genome-wide association study and genomic selection for yield and related traits in soybean
Source: PLoS One. 2021 Aug 13;16(8):e0255761. doi: 10.1371/journal.pone.0255761 (PMC8362977; doi:10.1371/journal.pone.0255761)

## Slide 1
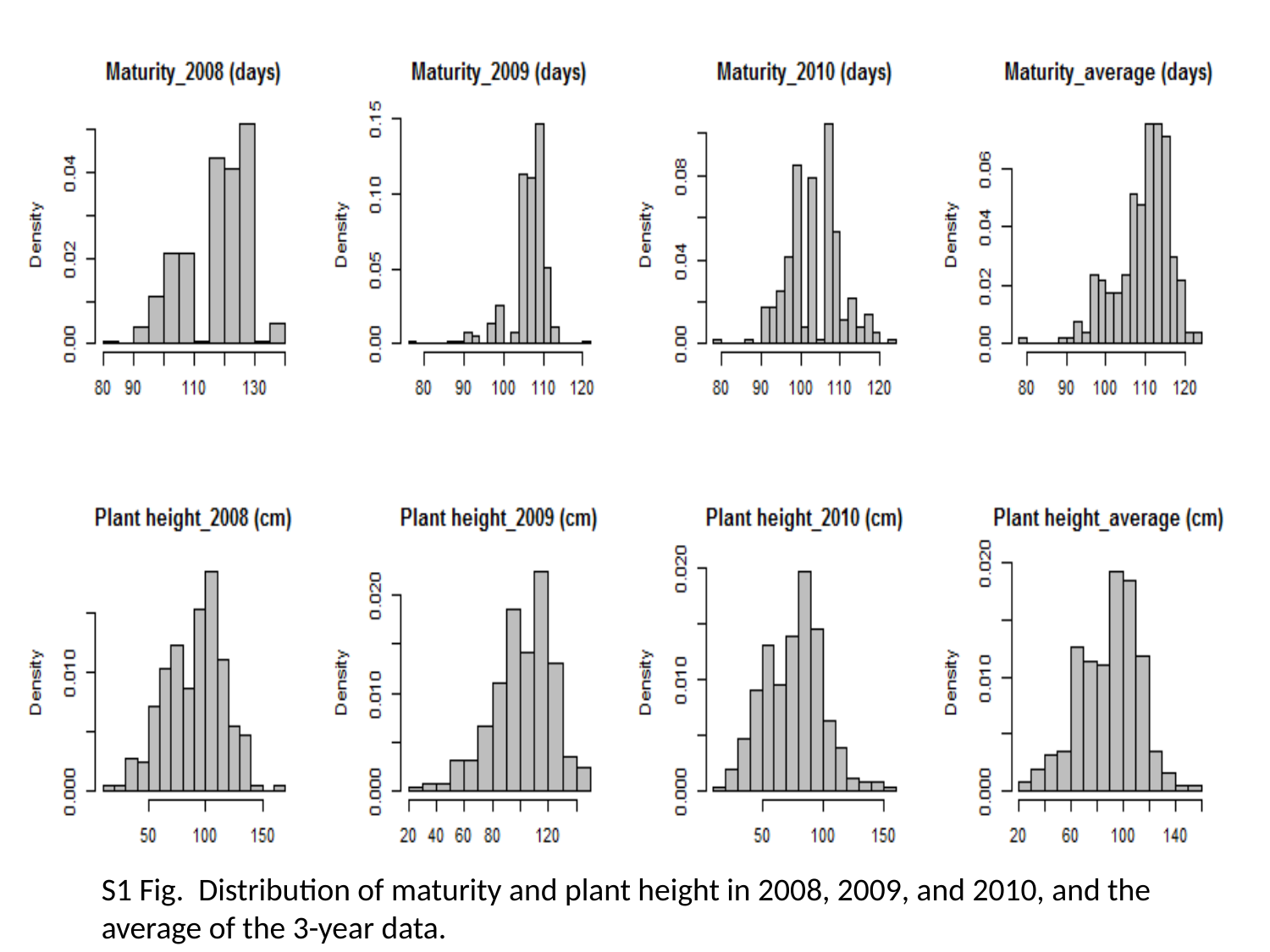

S1 Fig. Distribution of maturity and plant height in 2008, 2009, and 2010, and the average of the 3-year data.

Supplement: S1 Fig — (PPT) [file pone.0255761.s001.ppt]

## Slide 1
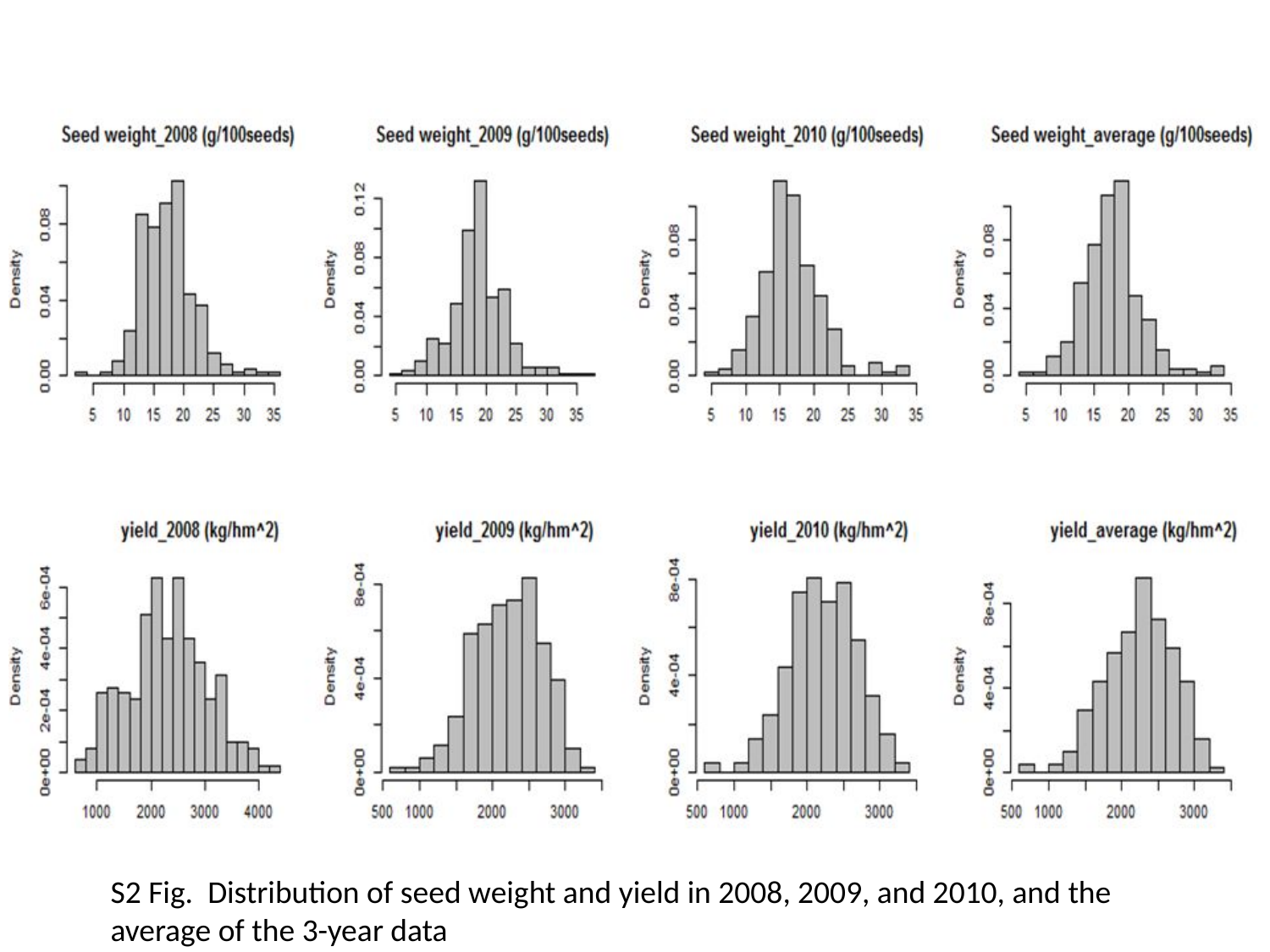

S2 Fig. Distribution of seed weight and yield in 2008, 2009, and 2010, and the average of the 3-year data

Supplement: S2 Fig — (PPT) [file pone.0255761.s002.ppt]

## Slide 1
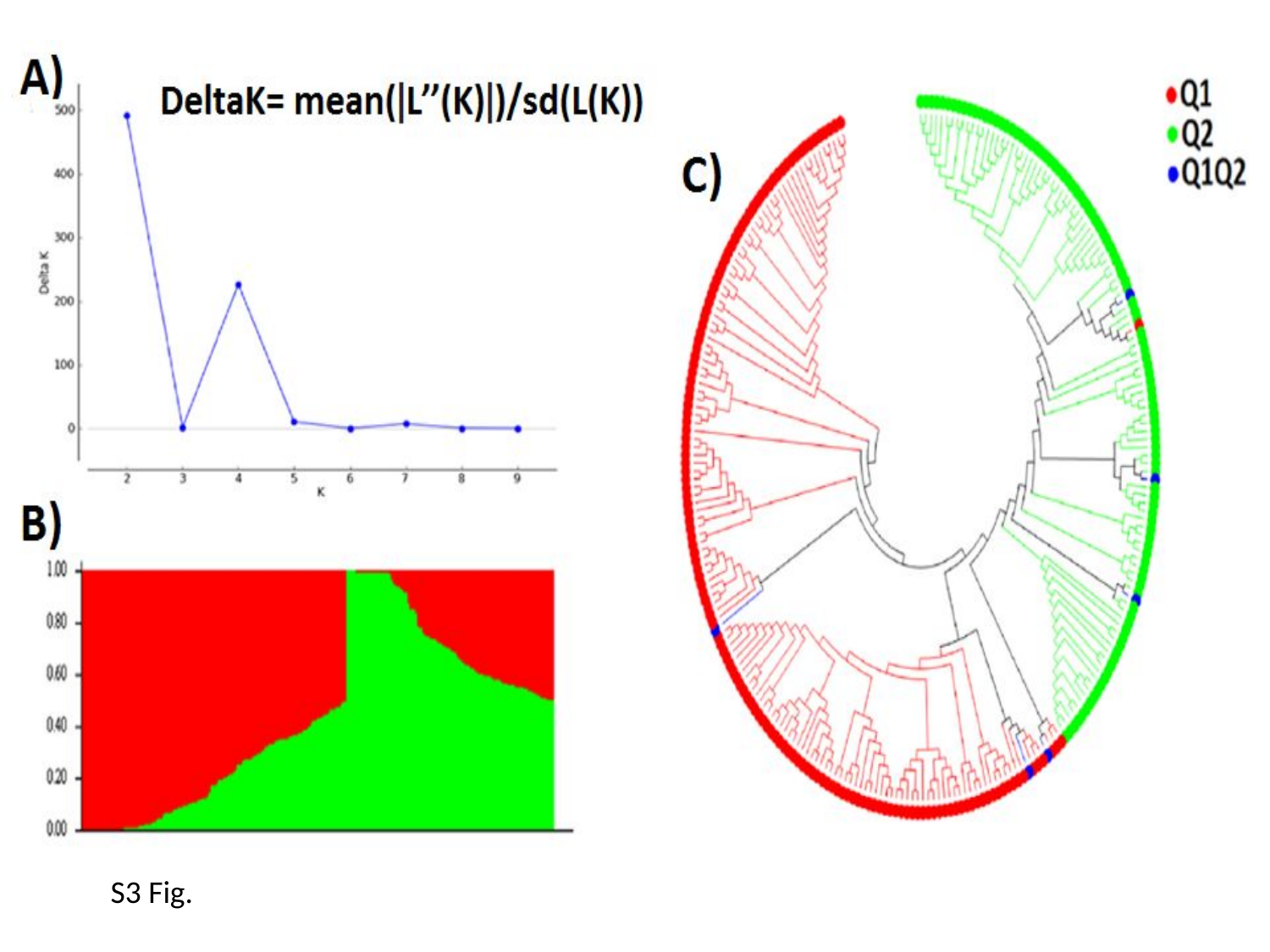

S3 Fig.

Supplement: S3 Fig — (A) Plot showing the delta K values on the y-axis and the corresponding K values on the x-axis. The plot was obtained from STRUCTURE Harvester (Earl and VonHoldt, 2011; http://taylor0.biology.ucla.edu/structureHarvester/). The delta K peak corresponds to K = 2. (B) Bar plot showing the population structure using STRUCTURE 2.3.4 (Pritchard et al. 2000) where the red color corresponds to cluster 1 and the green one to cluster 2. The y-axis of the bar plot indicates the proportion of membership of a genotype to each cluster. (C) Phylogenetic tree involves a combined analysis between population structure and genetic diversity. The solid red circles is for subpopulation 1 and the solid green circles is for subpopulation 2, solid blue circles is for admixture. (PPT) [file pone.0255761.s003.ppt]

## Slide 1
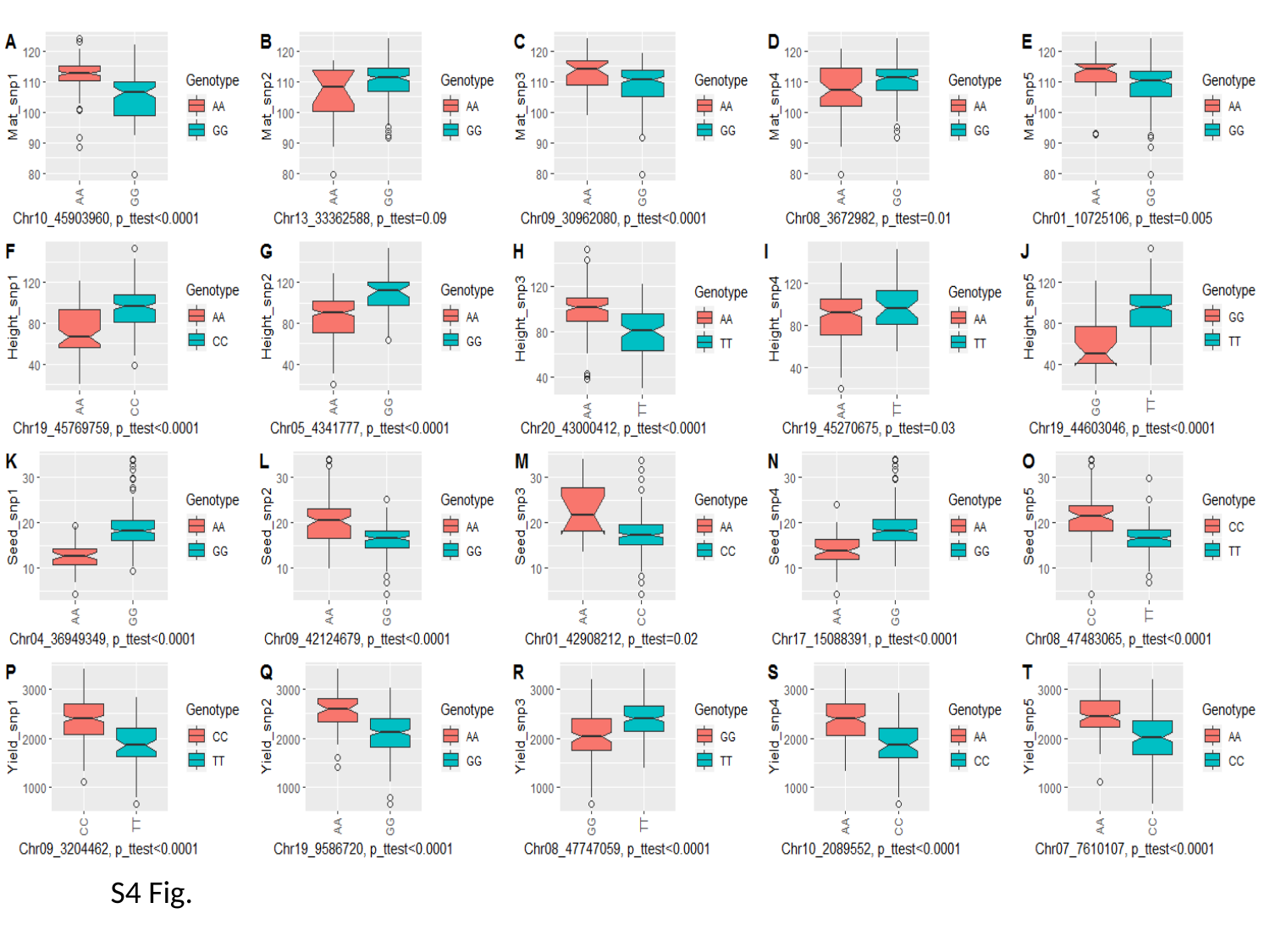

S4 Fig.

Supplement: S4 Fig — Manhattan plots and QQ-plots for yield in 2008 (A), 2009 (B), 2010 (C), and the combined data over 3 years (D). (PPT) [file pone.0255761.s004.ppt]

## Slide 1
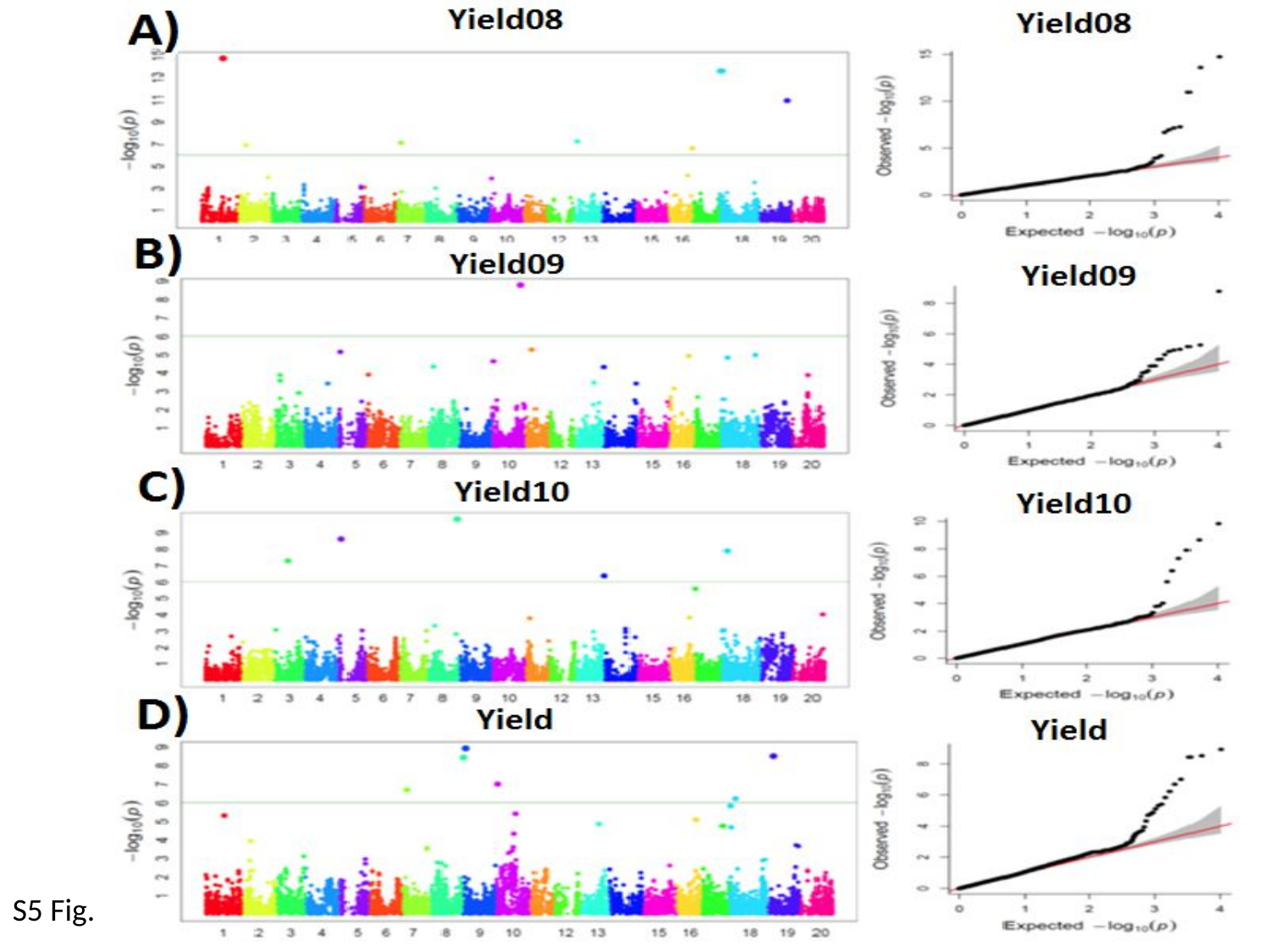

S5 Fig.

Supplement: S5 Fig — Boxplots showing the variation in plant maturity (A, B, C, D, and E), plant height (F, G, H, I, and J), 100-seed weight (K, L, M, N, and O), and grain yield (P, Q, R, S and T) within each genotypic class defined by the top 5 significant SNPs for each trait. The x-axis showed the genotypic class from each SNP, the y-axis showed the phenotypic value for each trait. On the y-axis, mat_snp, height_snp, seed_snp, and yield_snp denotes the maturity date, plant height, 100-seed weight and grain yield, respectively. (PPT) [file pone.0255761.s005.ppt]

## Slide 1
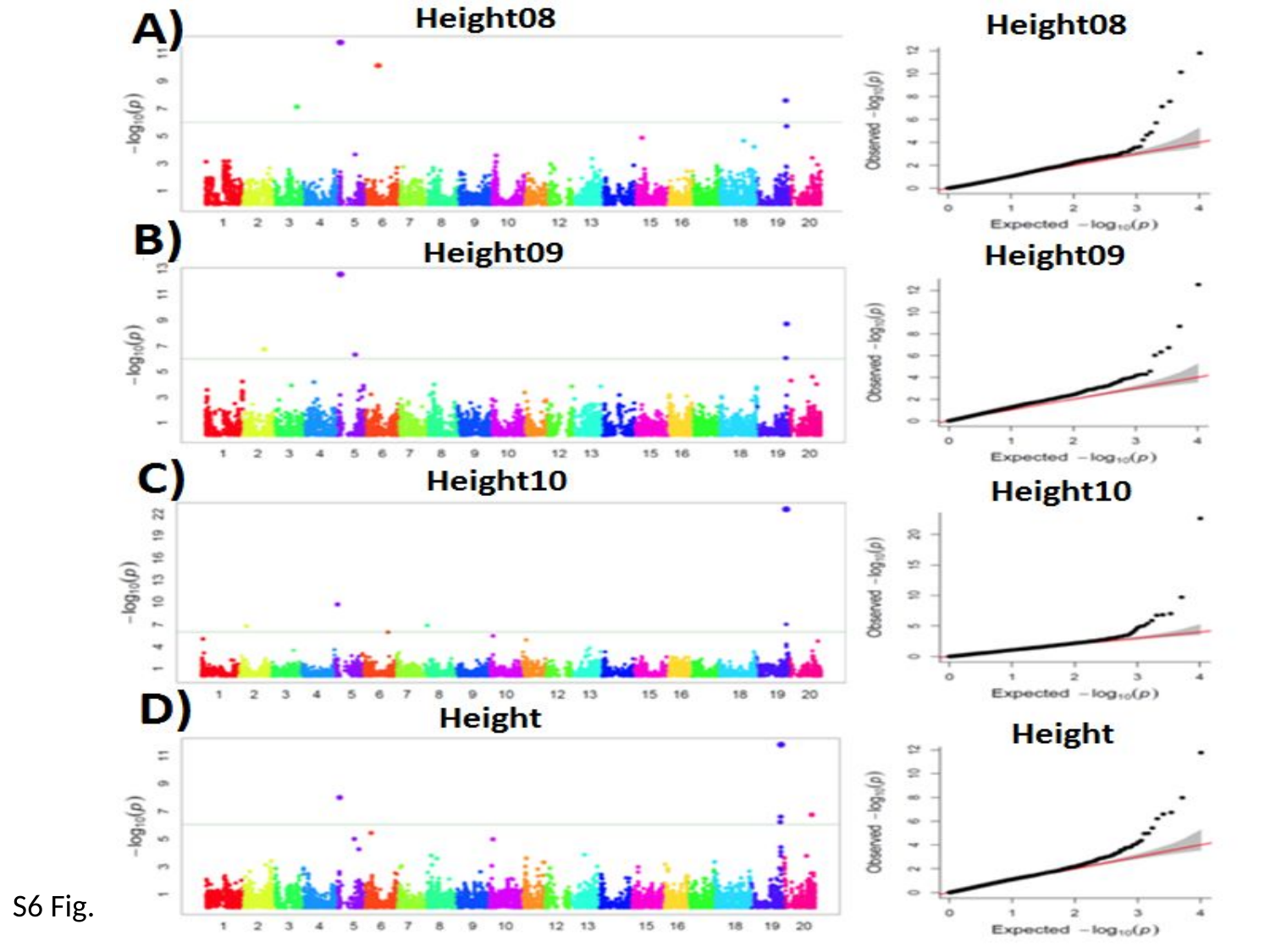

S6 Fig.

Supplement: S6 Fig — Manhattan plots and QQ-plots for plant height in 2008 (A), 2009 (B), 2010 (C), and the combined data over 3 years (D). (PPT) [file pone.0255761.s006.ppt]

## Slide 1
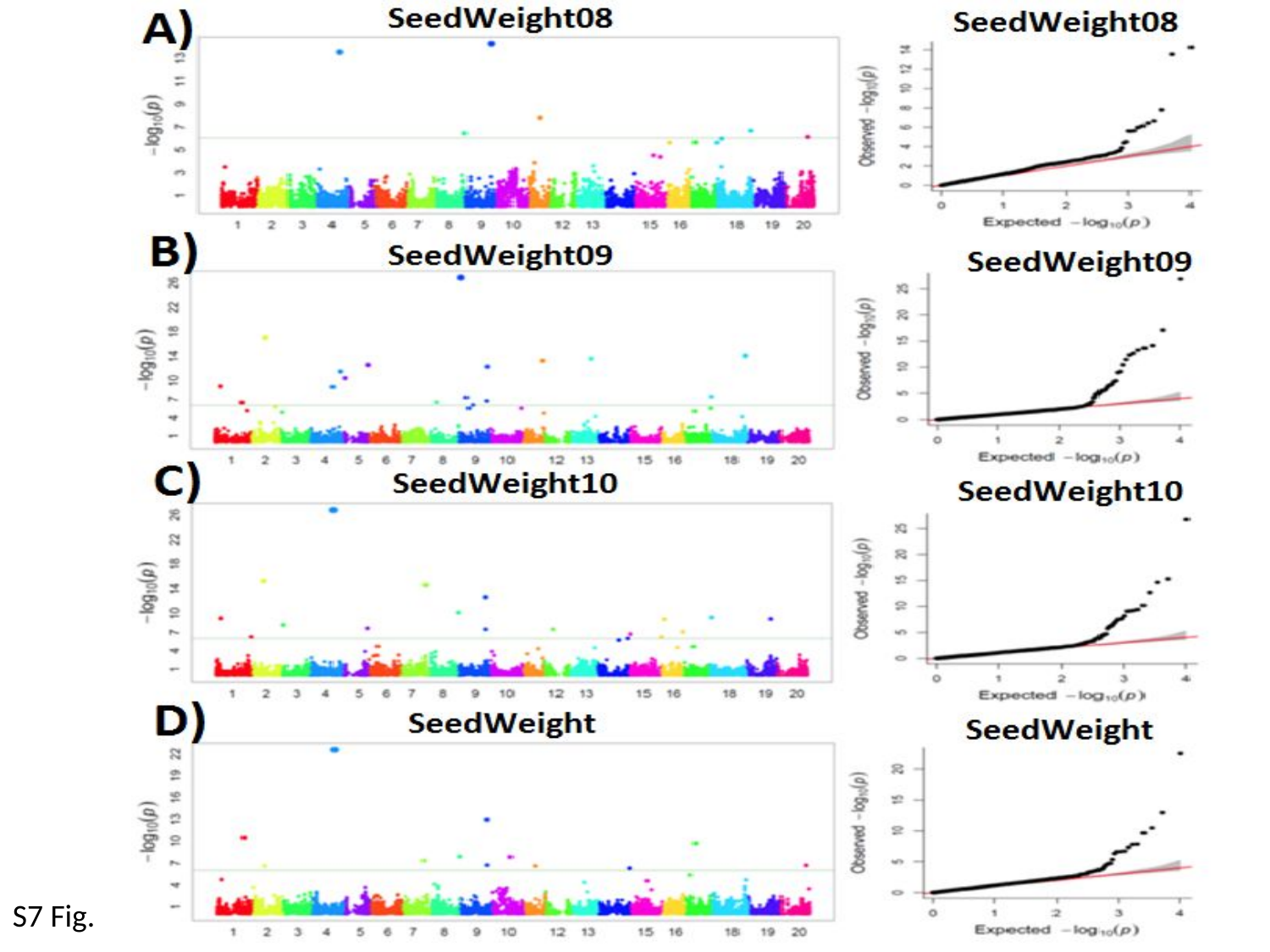

S7 Fig.

Supplement: S7 Fig — Manhattan plots and QQ-plots for seed weight in 2008 (A), 2009 (B), 2010 (C), and the combined data over 3 years (D). (PPT) [file pone.0255761.s007.ppt]

## Slide 1
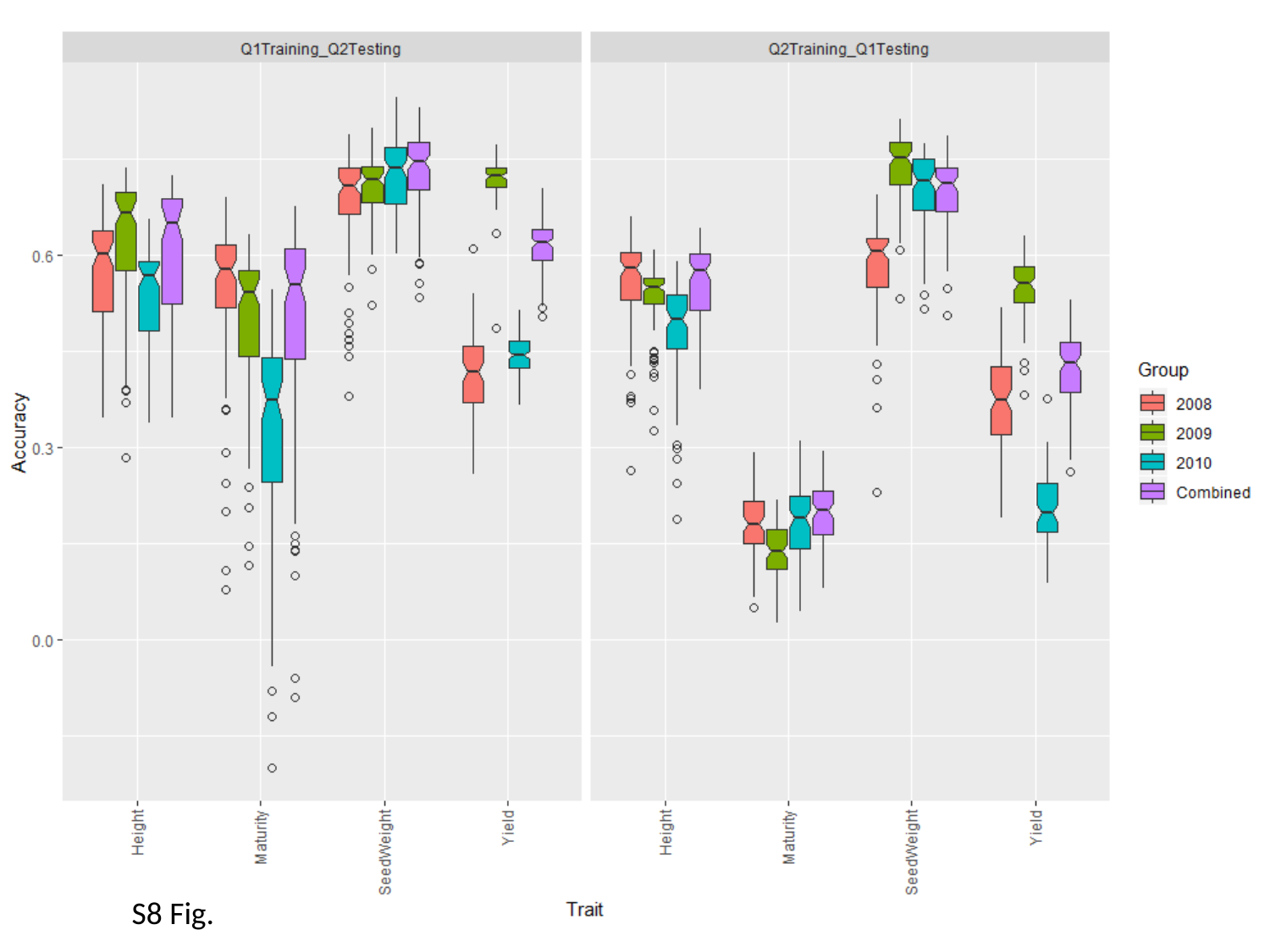

Fig. 10
S8 Fig.

## Slide 2
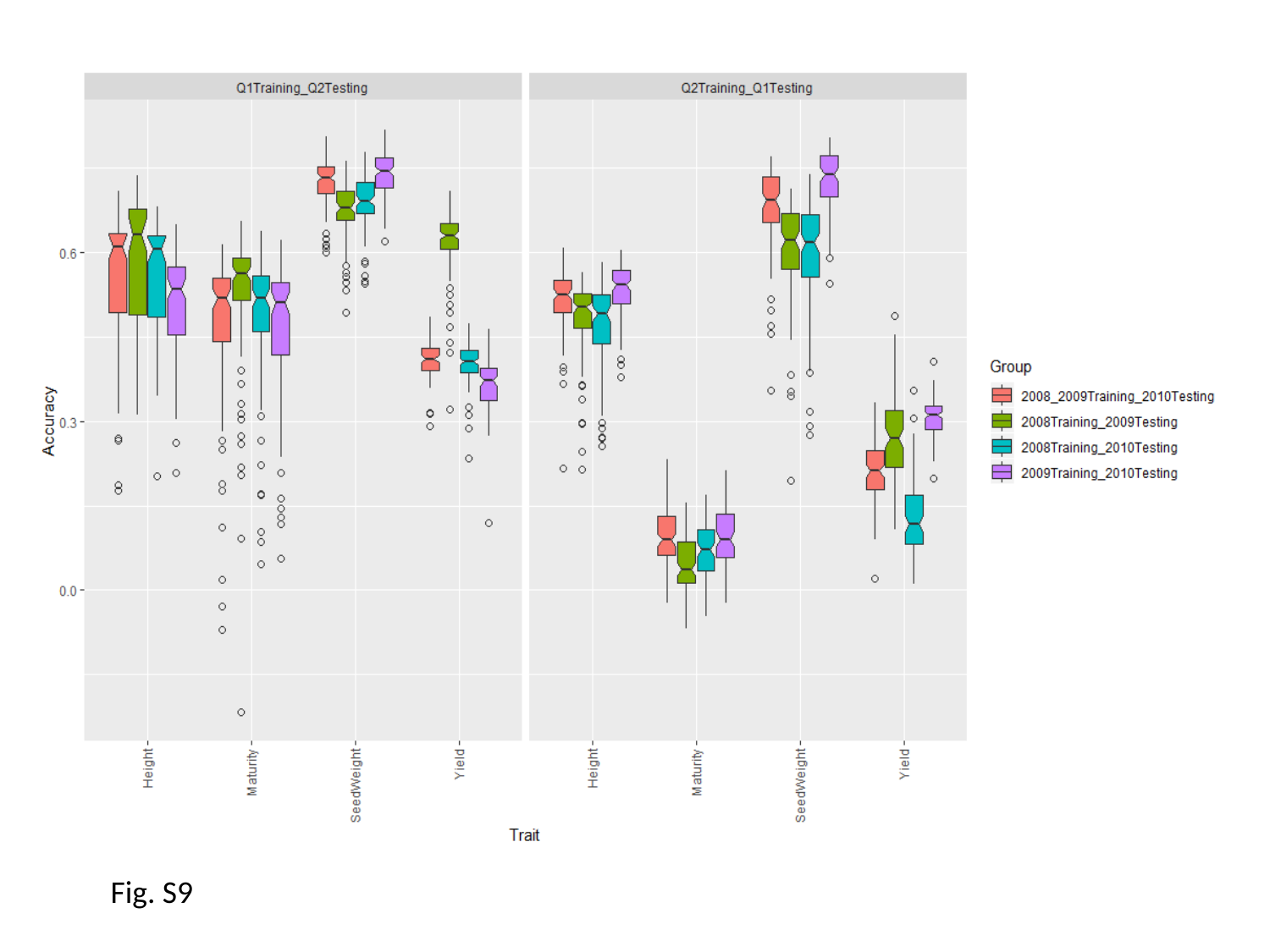

Fig. S9

Supplement: S8 Fig — Cross-validation was done using data from the same year. (PPT) [file pone.0255761.s008.ppt]

## Slide 1
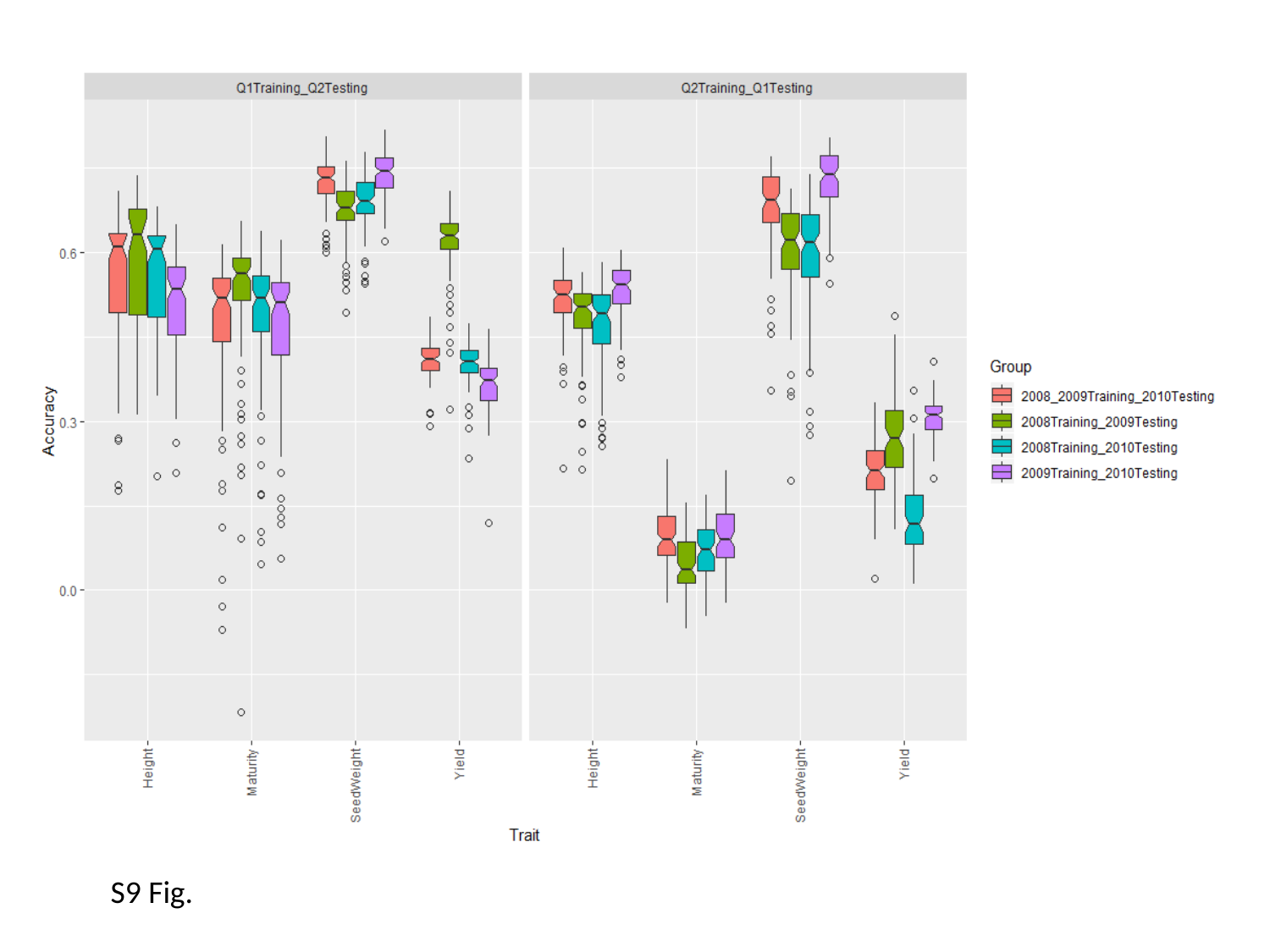

S9 Fig.

Supplement: S9 Fig — Cross-validation was performed using the data from a year to predict that of from the succeeding year(s). (PPT) [file pone.0255761.s009.ppt]
